# Decidualization of human endometrial stromal cells requires steroid receptor coactivator-3

**DOI:** 10.3389/frph.2022.1033581

**Published:** 2022-11-24

**Authors:** Vineet K. Maurya, Maria M. Szwarc, David M. Lonard, William E. Gibbons, San-Pin Wu, Bert W. O’Malley, Francesco J. DeMayo, John P. Lydon

**Affiliations:** ^1^ Department of Molecular and Cellular Biology, Baylor College of Medicine, Houston, TX, United States; ^2^ Department of Obstetrics and Gynecology, Baylor College of Medicine, Houston, TX, United States; ^3^ Reproductive and Developmental Biology Laboratory, National Institute of Environmental Health Sciences, Research Triangle Park, Durham, NC, United States

**Keywords:** steroid receptor coactivator-3, human endometrium, stromal cells, decidualization, RNA-sequencing

## Abstract

Steroid receptor coactivator-3 (SRC-3; also known as NCOA3 or AIB1) is a member of the multifunctional p160/SRC family of coactivators, which also includes SRC-1 and SRC-2. Clinical and cell-based studies as well as investigations on mice have demonstrated pivotal roles for each SRC in numerous physiological and pathophysiological contexts, underscoring their functional pleiotropy. We previously demonstrated the critical involvement of SRC-2 in murine embryo implantation as well as in human endometrial stromal cell (HESC) decidualization, a cellular transformation process required for trophoblast invasion and ultimately placentation. We show here that, like SRC-2, SRC-3 is expressed in the epithelial and stromal cellular compartments of the human endometrium during the proliferative and secretory phase of the menstrual cycle as well as in cultured HESCs. We also found that SRC-3 depletion in cultured HESCs results in a significant attenuation in the induction of a wide-range of established biomarkers of decidualization, despite exposure of these cells to a deciduogenic stimulus and normal progesterone receptor expression. These molecular findings are supported at the cellular level by the inability of HESCs to morphologically transform from a stromal fibroblastoid cell to an epithelioid decidual cell when endogenous SRC-3 levels are markedly reduced. To identify genes, signaling pathways and networks that are controlled by SRC-3 and potentially important for hormone-dependent decidualization, we performed RNA-sequencing on HESCs in which SRC-3 levels were significantly reduced at the time of administering the deciduogenic stimulus. Comparing HESC controls with HESCs deficient in SRC-3, gene enrichment analysis of the differentially expressed gene set revealed an overrepresentation of genes involved in chromatin remodeling, cell proliferation/motility, and programmed cell death. These predictive bioanalytic results were confirmed by the demonstration that SRC-3 is required for the expansion, migratory and invasive activities of the HESC population, cellular properties that are required *in vivo* in the formation or functioning of the decidua. Collectively, our results support SRC-3 as an important coregulator in HESC decidualization. Since perturbation of normal homeostatic levels of SRC-3 is linked with common gynecological disorders diagnosed in reproductive age women, this endometrial coregulator—along with its new molecular targets described here—may open novel clinical avenues in the diagnosis and/or treatment of a non-receptive endometrium, particularly in patients presenting non-aneuploid early pregnancy loss.

## Introduction

After embryo aneuploidy, parental chromosomal translocations, maternal thrombophilic anomalies, immunological disorders, and obvious uterine ultrastructural abnormalities are excluded as etiologic contributors, implantation failure intrinsic to the endometrium is commonly suspected as an underlying cause of early pregnancy loss [EPL (1)] and recurrent pregnancy loss [RPL (2)], the latter defined as the loss of two or more consecutive pregnancies in the first trimester (2–5). Accordingly, pregnancy success rates currently achieved by natural or assisted conception for women at high-risk for EPL or RPL can only be increased by an improved understanding of the cellular and molecular mechanisms that control endometrial function during the periimplantation period.

Soon after embryo attachment and the early stages of implantation, further invasion into the maternal compartment requires a functional decidua (6). Formation of the decidua entails progesterone-dependent transformation of endometrial stromal fibroblasts into specialized polygonal epithelioid decidual cells with ploidy; a process termed decidualization (7). Encapsulating the invading embryo, tightly adherent decidual cells form a decidual matrix that supports the development of the hemi-allogeneic conceptus. Decidual support includes providing histotrophic nutrition, protecting against physiological stressors, and promoting an immunotolerant microenvironment. Superimposed on these functions, the human decidua acts as a biosensor in which non-viable embryos are negatively selected (8, 9). Moreover, the decidua recruits innate immune cells [particularly uterine natural killer (uNK) cells (10)] to control the orderly invasion of the trophoblast into the maternal compartment and to orchestrate spiral arteriole remodeling that is essential for normal uteroplacental perfusion (11). In addition, uNK cells target and clear acute senescent decidual cells from the mature decidual cell population to maintain a healthy decidua (12).

During the first trimester, the above decidual functions emerge early in a series of cellular and molecular events that sequentially progress in the endometrium to advance the implanting embryo to hemochorial placentation. Accordingly, inadequate decidualization not only causes fetal demise at an early stage of gestation but is linked to a broad range of gestational complications that manifest in subsequent trimesters; these include early fetal miscarriage due to placental insufficiency, placenta accreta, fetal growth restriction, preeclampsia, and pre-term birth (13). Therefore, identifying new molecular signals that are essential for decidualization is critical to furnishing novel mechanistic insights that may lead to the development of more efficacious mechanism-based molecular diagnostics and/or precision therapies to improve the outcome of natural pregnancies as well as pregnancies conceived through assisted reproductive technologies.

The steroid receptor coactivator (SRC)/p160 family is composed of three pleiotropic coactivators (SRC-1, SRC-2, and SRC-3) (14), also known as NCOA1, NCOA2, and NCOA3 respectively. Members of the SRC/p160 family control a broad spectrum of physiologies that include (but are not limited to) metabolism, circadian rhythms, immunology, and parturition (15–20). Such SRC multifunctionality is attained through a complex functional domain structure, encompassing diverse protein-protein interaction regions that are highly responsive to posttranslational modifications (PTMs) (21, 22). Originally discovered as coactivators for nuclear receptor mediated signaling (23), SRCs are now known to control numerous non-nuclear receptor signaling pathways (14, 24). As documented for other physiological systems, members of the SRC family have been shown to play important roles in uterine biology and pathobiology (24). Here, we report that SRC-3 is critical for decidualization of human endometrial stromal cells (HESCs) in culture. Apart from a block in the signature morphological cellular changes that normally accompany HESC decidualization, SRC-3 depletion results in a markedly diminished induction of the majority of molecular biomarkers implicated in the decidualization process. Analysis of RNA profiling experiments underscores the importance of SRC-3 as a transcriptional coregulator that enables the pre-decidual HESC to appropriately respond to the deciduogenic stimulus through coregulator support of a genome-wide transcriptional program.

## Materials and methods

### Human endometrial tissue and immunohistochemistry

Using a pipelle suction curette, human endometrial biopsies were collected under sterile conditions from the uterine fundus of healthy women of reproductive age (27–38 years old). Participants had a normal uterus as evaluated by transvaginal ultrasound and were not receiving hormone treatment for at least 3 months before tissue biopsy. Endometrial tissue was biopsied during the proliferative (*n* = 6) or secretory (*n* = 5) phases of the cycle. The timed cycle phase was determined by the study participants using home ovulation test kits, and cycle stage of the resultant biopsy tissue was confirmed by histological analysis (25–27). Written informed consent was provided by the volunteers before the biopsy procedure, which was conducted in accordance with a protocol prospectively approved by the Institutional Review Board (IRB) at Baylor College of Medicine and in accordance with the guidelines of the Declaration of Helsinki (28).

For immunohistochemical analyses, tissues were fixed overnight in 4% paraformaldehyde in phosphate-buffered saline (PBS) before paraffin embedding and sectioning onto slides. Immunohistochemical detection of SRC-3 was achieved using a primary rabbit monoclonal antibody against human SRC-3 [Cell Signaling Technology Inc., Danvers, MA (#2126); diluted 1 : 400] followed by incubation with a horseradish peroxidase (HRP)-conjugated goat anti-rabbit antibody [Vector Laboratories Inc., Burlingame CA (*P*-1,000); diluted 1 : 200]. Peroxidase activity was detected with the Vectastain Elite ABC-HRP kit (Vector Laboratories Inc.). Following immunostaining, tissue sections were counterstained with hematoxylin before applying Permount mounting solution (Fisher Scientific Inc. (SP 15–500) to affix coverslips.

### Mouse xenograft studies

As previously described (29), singly dispersed endometrial cells [SDECs (containing stromal and glandular epithelial cells)] were prepared from human endometrial tissue biopsied during the proliferative phase of the cycle (*n* = 6). Using a Hamilton microliter syringe [Hamilton Company, Reno, NV (#95-901)], 5 × 10^5^ SDECs in 5–10 ul of sterile Dulbecco's Modified Eagle Medium (DMEM) were injected beneath the renal capsule of both kidneys of ovariectomized scid-beige immunocompromised host mice [Taconic Biosciences Inc., Rensselaer, NY (#CBSCBG)]; *n* = 3 per biopsy sample. Two weeks prior to injection, host mice were ovariectomized and subcutaneously implanted within the intrascapular region with a 90 day slow release pellet containing (1.5 mg 17 β-estradiol (E2)/pellet; Innovative Research of America Inc. Sarasota FL (#E-121)) (29). For E2 plus progesterone (P4) treatment, host mice were treated with E2 for 8 weeks before receiving a daily injection of P4 (1 mg P4/100 ul sesame oil) for 14 days. Following hormone treatment, reconstituted human endometrial tissue engrafted within the murine kidney capsule were fixed and processed for immunohistochemical analysis as described above.

Accredited by AAALAC (Association for the Assessment and Accreditation of Laboratory Animal Care), scid beige host mice were housed in a germ-free facility in the *vivarium* at Baylor College of Medicine, which is operated and controlled by the Center for Comparative Medicine. Studies with mice followed the guidelines detailed in the Guide for the Care and Use of Laboratory Animals [published by the National Research Council (Eighth Edition 2011)]. Prior to conducting experiments, animal research protocols were approved by the Institutional Animal Care and Use Committee (IACUC) at Baylor College of Medicine.

### Cell culture

The immortalized human endometrial stromal cell (T-HESC) line was obtained from the American Type Culture Collection [ATCC (CRL4003)] and maintained in phenol-red free DMEM/F12 medium supplemented with 10% charcoal/dextran-treated (stripped) FBS (sFBS: Sigma-Aldrich Inc., St. Louis, MO), 1% ITS-A (insulin, transferrin, selenite and sodium pyruvate), 500 ng/ml puromycin, 100 units/ml penicillin, and 0.1 mg/ml streptomycin (ThermoFisher Scientific Inc., Waltham, MA); medium was changed every other day. The authenticity of the T-HESC line was confirmed by short tandem repeat (STR) profiling by the ATCC cell line authentication service.

### Transfection of small interfering RNAs and *in-vitro* decidualization

Cells were cultured in six-well plates in triplicate before transfection with sixty picomoles of the non-targeting [*NT* (scrambled control sequence)] siRNA [GE Healthcare Dharmacon Inc., Lafayette, CO (D-001810-10-05)] or siRNA targeting *SRC-3* [Dharmacon Inc., (L-00 3759-00-0005)], using the Lipofectamine RNAiMAX transfection reagent (Invitrogen Inc., Carlsbad CA) (30). Forty-eight hours post-transfection of siRNAs, T-HESCs were cultured in deciduogenic medium to stimulate decidualization (100 nM 17β-estradiol [Sigma-Aldrich Inc. (E1024)], 10 μM medroxyprogesterone acetate (MPA [Sigma-Aldrich Inc. (M1629)], and 50 μM N6, 2′-O-dibutyryladenosine 3′, 5′ cyclic monophosphate sodium salt [Sigma-Aldrich Inc. (D0260)] in 1× Opti-MEM reduced serum medium, containing 2% charcoal-stripped FBS (hereon referred to as EPC medium); EPC medium was changed every 48 h.

### Immunocytochemistry

After transfection with either *NT* or *SRC-3* siRNAs, T-HESCs were cultured in EPC medium on coverslips coated with poly-L-lysine [Sigma-Aldrich Inc. (P4707)]. Following a specified time period of culture, T-HESCs were fixed on coverslips with 4% paraformaldehyde in PBS for 15 min at room temperature. Following fixation, cells were washed three times with PBS before permeabilization with 0.1% Triton X-100 in PBS at room temperature for 20 min. Permeabilized cells were washed with PBS, then blocked with 2% bovine serum albumin in PBS for 1 h at room temperature before incubation overnight at 4°C with a primary antibody against human SRC-3 [Cell Signaling Technology, Inc. (#2126); 1 : 200 dilution]. After washing with PBS, T-HESCs were incubated with the Alexa Fluor 594-conjugated secondary antibody [Life Technologies, (A21207); 1 : 500 dilution] and Alexa Fluor 488 Phalloidin [Invitrogen Inc., (A12376)] for 1 h at room temperature, washed, and then mounted with Vectashield Antifade mounting medium with 4′, 6′-diamidino-2-phenylindole [DAPI; Vector Laboratories Inc., (H-1200-10)]. Raw images were captured using a color chilled AxioCam MRc5 digital camera attached to a Carl Zeiss AxioImager A1 upright microscope equipped for epifluorescence detection (Zeiss Inc., Jena, Germany). Post image processing and annotation for the purposes of data presentation were performed using the latest versions of the Photoshop and Illustrator software programs provided within Adobe Creative Suite (Adobe Systems Inc., San Jose CA).

### Quantitative real-time polymerase chain reaction

Cells were lysed in RNA lysis buffer before total RNA was isolated with the Purelink RNA Mini Kit [ThermoFisher Scientific Inc. (#12183020)]; the Nano-Drop 2000 UV/Visual spectrophometer (ThermoFisher Scientific Inc.) was used for RNA quantification. Total RNA (1 µg) was reverse transcribed using the High-Capacity cDNA Reverse Transcription Kit [ThermoFisher Scientific Inc. (#4368814)]. Amplified cDNA was diluted to a concentration of 10 ng/µl before quantitative real time PCR (qPCR) was performed; qPCR was performed using the Fast TaqMan 2X Mastermix (Applied Biosystems/Life Technologies, Grand Island, NY). The TaqMan assays used in this study are listed in Table 1. All qPCR experiments were performed using the 7,500 Fast Real-time PCR system (Applied Biosystems/Life Technologies, Grand Island, NY); the delta-delta cycle threshold was used to normalize expression to the 18S reference.

**Table 1:** List of human taqman expression assays used in these studies.

Gene	ID	Catalog number
CCN2	1,490	Hs00170014_m1
CCN3	4,856	Hs00159631_m1
IGFBP1	3,484	Hs00236877_m1
H2AC21	317,772	Hs00602439_s1
H3C1	8,350	Hs00543854_s1
H3C7	8,968	Hs00851863_s1
H3C10	8,357	Hs00818527_s1
H4C3	8,364	Hs00543883_s1
H4C4	8,360	Hs00371424_s1
HAND2	9,464	Hs00232769_m1
HOXA10	3,206	Hs00172012_m1
INHBA	3,624	Hs01081598_m1
PGR	5,241	Hs01556702_m1
PRL	5,617	Hs00168730_m1
SCARA5	286,133	Hs01073151_m1
SST	6,750	Hs00356144_m1
NCOA1	8,648	Hs00186661_m1
NCOA2	10,499	Hs00896109_m1
NCOA3	8,202	Hs00180722_m1
TIMP1	7,076	Hs01092512_g1
WNT4	54,361	Hs01573504_m1
18S rRNA	ThermoFisher Scientific Inc.	4319413E

### Genome-wide RNA expression profiling

Genome-wide RNA-sequencing (RNA-seq) and analysis were performed as described (30, 31). Briefly, total RNA purity and integrity were assessed using the NanoDrop spectrophotometer (ThermoFisher Scientific Inc.) and the 2,100 Bioanalyzer with RNA chips (Agilent Technologies, Santa Clara, CA) respectively. Only RNA samples scoring a RNA integrity number (RIN) of 8 or greater were included in subsequent RNA profiling experiments. For each experimental group, RNA samples from two replicates were used. Total RNA was reverse transcribed using the Ovation RNA-seq System V2 [Tecan Genomics Inc., Redwood City CA (#7102-32)]. For these experiments, cDNA libraries were generated with the Nextera DNA Flex Library Prep and the Nextera DNA CD indexes Kits [Illumina Inc., San Diego CA (#20018704)]. Using the Illumina NovaSeq 6,000 system, cDNA libraries were sequenced to a 50-nucleotide read length in the paired end format. Raw reads were processed using the Partek Flow Genome Analysis Software [Partek Inc., St. Louis, MO (version 10.0.21.0801)]. Reads for ribosomal and mitochondrial DNAs were removed by Bowtie 2 software (version 2.2.5), followed by base trimming with reference to quality scores with default parameters. Read alignment to the human genome hg38 was carried out using HISAT2 software (version 2.1.0). Both singleton and unaligned reads were removed using the default parameters set within the Partek Flow Genomics Analysis Software (Partek Inc.). Aligned and filtered reads were quantified to the National Center for Biotechnology Information RefSeq Transcripts 100 (2021-11-01), using the quantify-to-annotation model [(Partek E/M) function with default parameters] in Partek Flow (Partek Inc.). Gene counts were normalized to fragments per kilobase of exon per million mapped fragments (FPKM). For hierarchical clustering, the pheatmap package in R was used to draw the clustered heatmap based on FPKM values. Principal component analysis (PCA) and lists of differentially expressed genes were generated by the Partek Genomics Suite 7.0 (Partek Inc.).

The Bioconductor package EdgeR identified differentially expressed genes between the control and test group (32). The false discovery rate (FDR) of differentially expressed genes between the two groups was determined by Benjamini and Hochberg analysis (33). Differential gene expression between the two groups with a FDR value ≤0.05 and an absolute fold change (IFCI) ≥ 1.5 was considered significant and used further to identify affected pathways (34). Gene ontology enrichment analysis was conducted using the DAVID (Database for Annotation, Visualization, and Integrated Discovery) functional annotation clustering tool (http://david.abcc.ncifcrff.gov/) (35). Overrepresented established gene sets were identified by Gene Set Enrichment Analysis (GSEA; http://software.broadinstitute.org/gsea/) (36, 37). Hallmark gene sets from the Molecular Signatures Database (MSigDB) were used in these GSEA studies (38).

### Immunoblotting

Protein (20 μg) from cell lysates was resolved on 10% or 4%–15% sodium dodecyl sulfate-polyacrylamide (SDS-PAGE) gels before transfer to polyvinylidene difluoride (PVDF) membranes. After protein transfer, PVDF membranes were blocked for 1 h with 5% non-fat dry milk [Santa Cruz Biotechnology Inc., Dallas, TX; (# SC-2324)] in Tris-buffered saline with Tween 20 (TBS-T) and incubated overnight at 4°C with the following primary antibodies: anti-SRC-3 [Cell Signaling Technology, Inc., (#2126)] diluted 1:1,000 and anti-β-actin [Novus Biologicals, Piscataway, NJ; (#NB10056874T)] diluted 1:10,000 in 5% non-fat milk in TBS-T. Immunoblots were then probed with anti-rabbit IgG secondary antibody (ThermoFisher Scientific Inc.; [#A27036 (1:5,000 dilution)] or anti-mouse IgG [Cell Signaling Technology, Inc.; #7076 (1:10,000 dilution)] secondary antibodies conjugated with HRP in 5% non-fat milk in TBS-T for 1 h at room temperature. The following primary antibodies were used to detect SRC-1 (anti-human SRC-1 rabbit monoclonal; Cell Signaling Technology Inc., #2191) and SRC-2 (anti-human SRC-2 mouse monoclonal antibody; BD Biosciences Inc. #610984). The primary antibody against the phospho-serine (S857) residue in human SRC-3 was obtained from Cell Signaling Technologies Inc. (#P57249 PP5). Chemiluminescence was detected with the SuperSignal West Pico PLUS Chemiluminescent Substrate (ThermoFisher Scientific, Inc.; #1863097). Immunoreactive bands were digitally imaged using the Azure 600 Imaging Systems (Azure Biosystems, Sierra Court, Dublin, CA). Densitometric analysis of immunoreactive bands corresponding to SRC-3 and β-actin (loading control) was performed using ImageJ software [version 1.53t (https://imagej.nih.gov)], a publically available Java-based image processing program.

### Cell proliferation/viability assay

At a density of 3 × 10^3^ cells per well, cells were seeded in 96-well culture plates in triplicate. Cells transfected with siRNAs for 48 h were further cultured for 0, 24, 48, 72 or 96 h before cell proliferation was measured using the CellTiter 96® Non-Radioactive Cell Proliferation Assay kit [Promega Inc. Madison, WI; (#G4000)] (30). Following a predetermined time period in culture, 15 μl of 3-(4, 5-dimethylthiazol-2-yl)-2, 5-diphenyltetrazolium bromide (MTT); Promega, Madison, WI) was added to each well to a final concentration of 0.5 mg/ml. In the dark, cells were then incubated at 37 °C for an additional three hours. Following supernatant removal, formazan crystals were dissolved by the addition of the stop/solubilizing solution {100 µl [dimethyl sulfoxide (DMSO)/well]}. The absorbance of the final mixture was recorded at 570 nm (the formazan absorbance maximum) using the Multiskan FC Microplate Photometer (Thermo Scientific Inc., #51119000). The mean absorbance at “*N*” time point/mean absorbance at 0 h (*N* = 24, 48, 72, and 96 h) calculated relative cell proliferation (30). Each experiment was repeated three times with at least three to five technical replicates for each treatment group.

### Cell migration assay

As previously described (30), comparative cell migration was quantified using the *in vitro* wound-healing assay. Cells were seeded in six-well culture plates and cultured to 70%–80% confluency prior to siRNA transfection. A 200-μl sterile pipette tip generated a linear scratch (wound) in the middle of the cell monolayer within each well. After gentle washing to remove non-adherent cells, digital images were captured using an inverted phase-contrast microscope [EVOS^TM^ XL Core Imaging System (ThermoFisher Scientific Inc. #AMEX1000)]. Following forty-eight hours of culture, the degree of wound closure per time point was recorded by digital image capture. The wound area was calculated by manual tracing the cell-free area within each captured image per experimental group using ImageJ software (https://imagej.nih.gov/ij/). Results were recorded as a percent of wound closure in comparison to control after a forty-eight hour culture period (percent cell migration area = wound width at 0 h – wound width at 48 h/wound width at 0 h). Each experiment was repeated three times with triplicates for each treatment group.

### Transwell cell invasion assay

The Corning BioCoat Matrigel Invasion Chamber Kit [ThermoFisher Scientific Inc. (#354480)] was used for cell invasion assays (30). Following the forty-eight hour siRNA transfection period, T-HESCs were suspended in Opti-MEM medium. To the bottom of each transwell of the invasion chamber plate, culture medium with 20% FBS was added (0.6 ml). To test migration potential, suspended cells (8 × 10^4^ cells/250 µl) were added to each transwell insert. After forty-eight hours, a cotton swab carefully removed cells on the upper surface of the transwell. Following fixation with 4% paraformaldehyde in PBS, migrated cells were stained with a crystal violet solution (30). Washed inserts were digitally imaged using a Zeiss stereo-microscope with an attached AxioCam MRC-5 digital camera (Zeiss Inc., Jena, Germany). Stained migrated cells were counted in four separate cell fields throughout the insert before an average number of migrated cells was calculated (30). Each experiment was repeated three times with triplicates for each treatment group.

### Statistical analysis

Two-tailed unpaired Student *t*-tests with Welch's correction were used to estimate the statistical significance of differences between control and test groups. One-way ANOVA was used for multiple comparisons to analyze experiments containing more than two groups. Unless otherwise stated, data were graphically presented as mean ± standard deviation (SD). Differences with *p*-values <0.05 were considered statistically significant; asterisks represent the level of significance: *p < 0.05, **p < 0.01, ***p < 0.001, and ****p < 0.0001. Prism software version 9 (GraphPad Software Inc., San Diego CA) was used for the majority of the reported statistical analyses.

## Results

### Steroid receptor coactivator-3 is expressed in human endometrial epithelial and stromal cells

**Figure 1:**
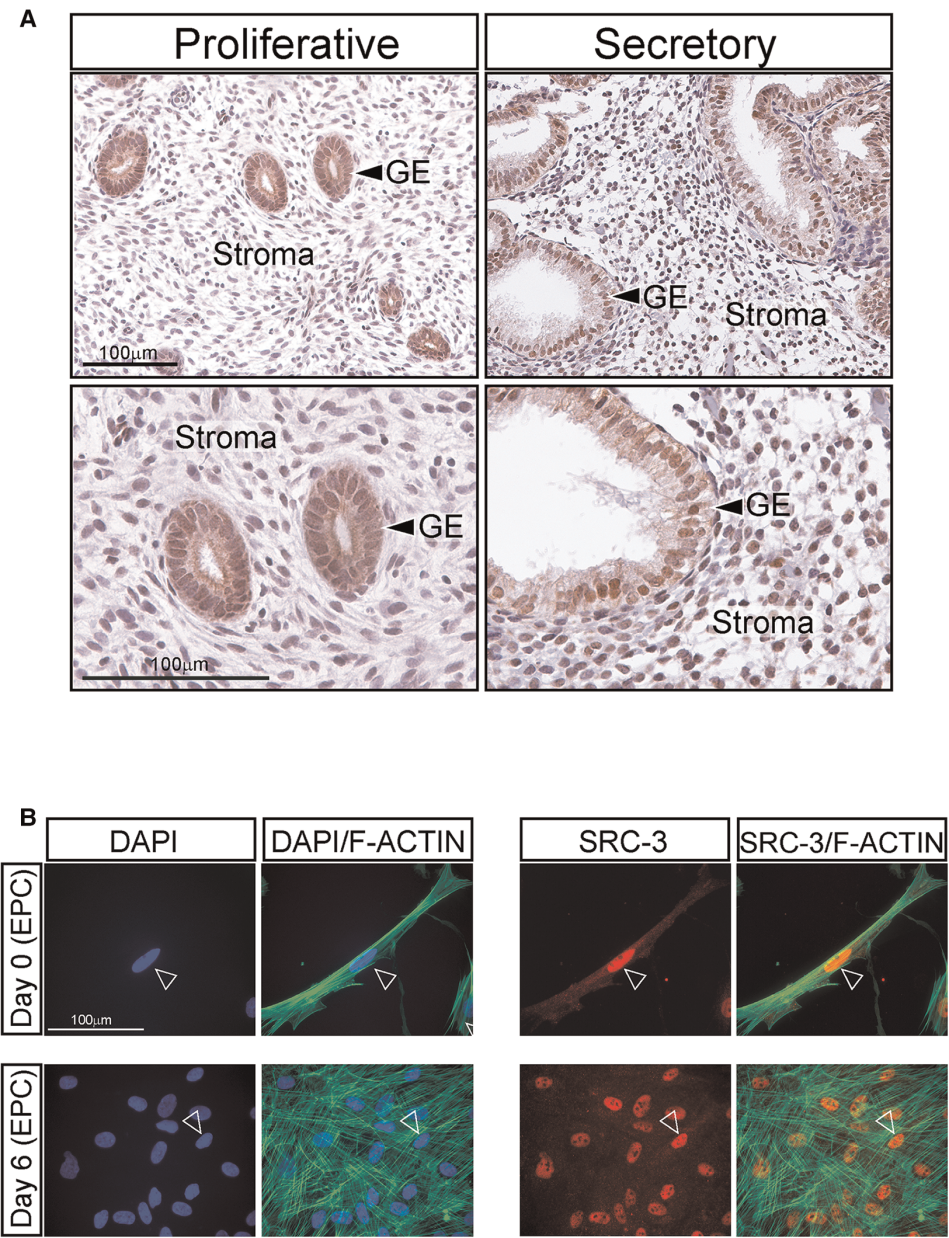
Expression of SRC-3 in human endometrial cells (**A**) left column of panels shows low and high power magnification images (top and bottom panels respectively) of human endometrial tissue biopsied during the estrogen-dominant proliferative phase of the menstrual cycle, which was immunohistochemically stained for SRC-3 expression. Note that most glandular epithelial (GE) cells express nuclear localized SRC-3 while stromal cells express lower levels of SRC-3 at this stage of the cycle. Right column of panels displays low and higher magnification images (top and bottom panels respectively) of human endometrial tissue biopsied during the progesterone-dominant secretory phase and stained for SRC-3 expression. Expression of nuclear SRC-3 is clearly detected in the GE cells, which exhibit a distinctive tortuous morphology at this stage of the cycle. Nuclear expression of SRC-3 in the stroma is clearly observed in the majority of cells. Scale bars in the top and bottom left panels apply to the top and bottom right panels respectively. (**B**) The left two columns show cultured T-HESCs stained with DAPI (blue nucleus) or DAPI (blue) plus phalloidin (green; binds F-actin) on day 0 and 6 of EPC treatment; arrowhead denotes nucleus of T-HESC. On day 0 of EPC treatment, T-HESCs exhibit a fibroblastic morphology with F-actin fibers orientated parallel to each other. Following six days of EPC treatment, note the conspicuous spatial disorganization of the F-actin fibers indicative of cellular transformation from a fibroblastoid to an epithelioid cell morphology (39). The two columns on the right show the same cells immunofluorescently stained for SRC-3 expression [red (arrowhead)] or SRC-3 (red) plus phalloidin (green). Note that SRC-3 is expressed in all T-HESCs at days 0 and 6 of EPC treatment with most of its expression localized to the nucleus (arrowhead). Scale bar applies to all panels.

Immunohistochemical analysis revealed that SRC-3 protein is expressed in nuclei of glandular epithelial and stromal cells of human endometrial tissues biopsied during the estrogen-dominant proliferative phase of the menstrual cycle (Figure 1A). In addition, SRC-3 expression was detected in the same endometrial cellular compartments during the progesterone-dominant mid-secretory phase of the cycle (Figure 1A). Although a moderate increase in endometrial epithelial and stromal SRC-3 expression is qualitatively observed during the secretory phase of the cycle, the overall expression profile of SRC-3 does not significantly change between the two cycle stages. These results indicate that estrogen and progesterone do not significantly control SRC-3 expression in the human endometrium. This conclusion is further supported by xenograft experiments that entailed using SDECs derived from human endometrial tissue biopsies transplanted beneath the renal capsule of ovariectomized immunocompromised scid beige mice, which were then treated with estrogen (E2) or estrogen plus progesterone (E2P4) (Supplementary Figure S1). Using this controlled hormone treatment model, the SRC-3 expression profile did not markedly change between E2 and E2P4 treatment (Supplementary Figure S1). The above conclusions generally agree with conclusions drawn from previous investigations that used a different primary SRC-3 antibody (40) or studies that investigated SRC-3 expression only at the RNA level (41). The expression of SRC-3 protein was also detected in nuclei of cultured T-HESCs (Figure 1B). The expression of SRC-3 was detected in all T-HESCs irrespective of whether these cells were in the pre-decidual ([Day 0 (EPC)] or decidualized [Day 6 (EPC)] state.

### Expression levels of SRC-3 in T-HESCs do not significantly change during the decidualization process

**Figure 2:**
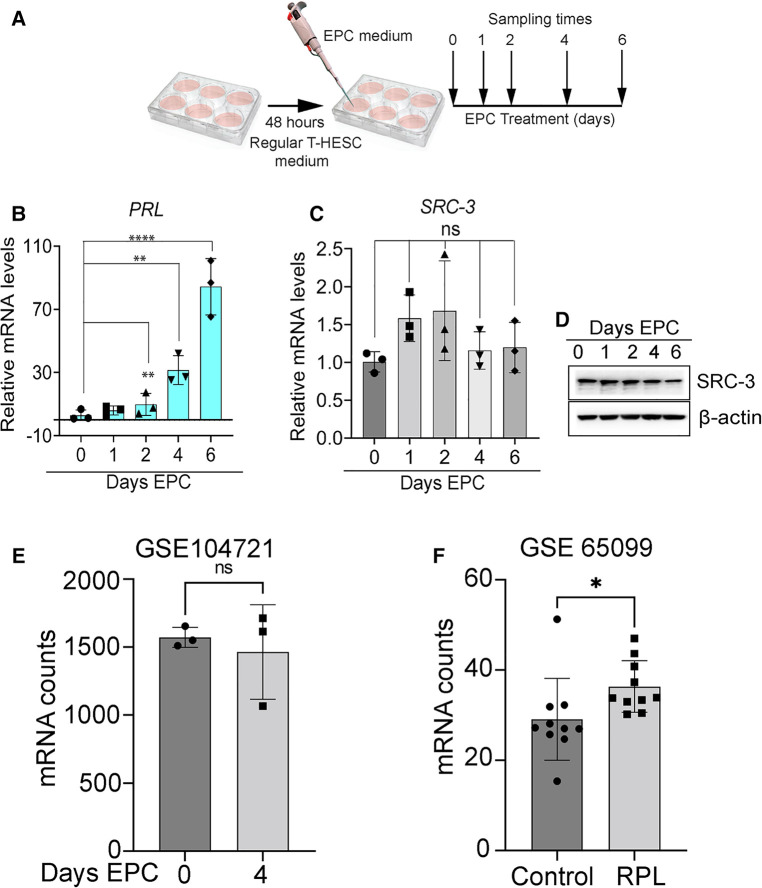
Expression levels of SRC-3 do not significantly change during T-HESC decidualization. (**A**) Schematic summarizes the experimental design of EPC decidualization of T-HESCs in culture. Following culture in standard culture medium for forty-eight hours, T-HESCs were switched to EPC medium to elicit decidualization over time. Cells were harvested at 0, 1, 2, 4, and 6 days following EPC administration for qPCR and western analysis. (**B**) As expected, the established *PRL* biomarker for decidualization (43) is significantly induced following EPC administration with time. (**C**) The expression levels of *SRC-3* do not markedly change from day 0 to day 6 of EPC treatment. (**D**) The results in (**C**) are confirmed at the protein level by western analysis, which show there is minimal change in the levels of SRC-3 protein during T-HESC decidualization. (**E**) From a publicly available GEO dataset [GSE: 104721 (44)], raw transcript scores for *SRC-3* (*NCOA3*) were calculated from HESCs cultured at day 0 (non-decidualized) and day 4 (decidualized) in deciduogenic medium. Note that SRC-3 transcript levels in T-HESCs do not significantly change following decidualization; *n* = 3 samples per treatment group. (**F**) The SRC-3 raw transcript scores from endometrial RNA obtained from women diagnosed with RPL compared with patients diagnosed with no RPL (control) (45); endometrial tissue was biopsied at the mid-secretory phase of the cycle. Original transcript data were obtained from the publically available RNA-seq dataset [GEO accession: GSE 65099 (45)]. Analysis reveals that SRC-3 RNA is significantly increased in endometrial tissue biopsied from RPL patients when compared to control patients. Results are displayed as the mean ± SE; *n* = 10 women per RPL and control groups.

Although the level of prolactin (*PRL*; an established decidual biomarker (42) significantly increases as T-HESCs decidualize with time, the level of SRC-3 expression does not markedly change (both at the RNA and protein level) during the time course of treatment with the deciduogenic hormone stimulus (EPC) (Figures 2A–D). These results also agree with expression data obtained from analyzing a publically available RNA-seq dataset (GEO accession: GSE104721) (44). This study used cultured primary HESCs either untreated (day 0 EPC) or treated with EPC for 4 days (44) (Figure 2E). Our analysis of this dataset shows that SRC-3 mRNA levels do not significantly change in primary HESCs either in the pre-decidual or decidualized state. While SRC-3 protein levels do not change in the pre-decidual and decidualized HESC, the phosphorylation status of SRC-3 changes in response to the EPC deciduogenic stimulus (Supplementary Figure S2). These results suggest that SRC-3 functional activity may be modulated by EPC-induced PTMs rather than control of the absolute levels of SRC-3 protein. Interestingly, analysis of separate publically available RNA-seq dataset (GEO accession: GSE65099) (45) showed that SRC-3 RNA levels are significantly increased in the endometrium of patients diagnosed with RPL (Figure 2F), suggesting that perturbation of the homeostatic levels of SRC-3 may be linked to this endometrial pathology. It's important to note that dysregulation of SRC-3 expression is known as a critical causal factor in the genesis and progression of numerous tissue pathologies, including tumorigenesis and metastasis (14, 46, 47).

### Decidualization of T-HESCs requires SRC-3

**Figure 3:**
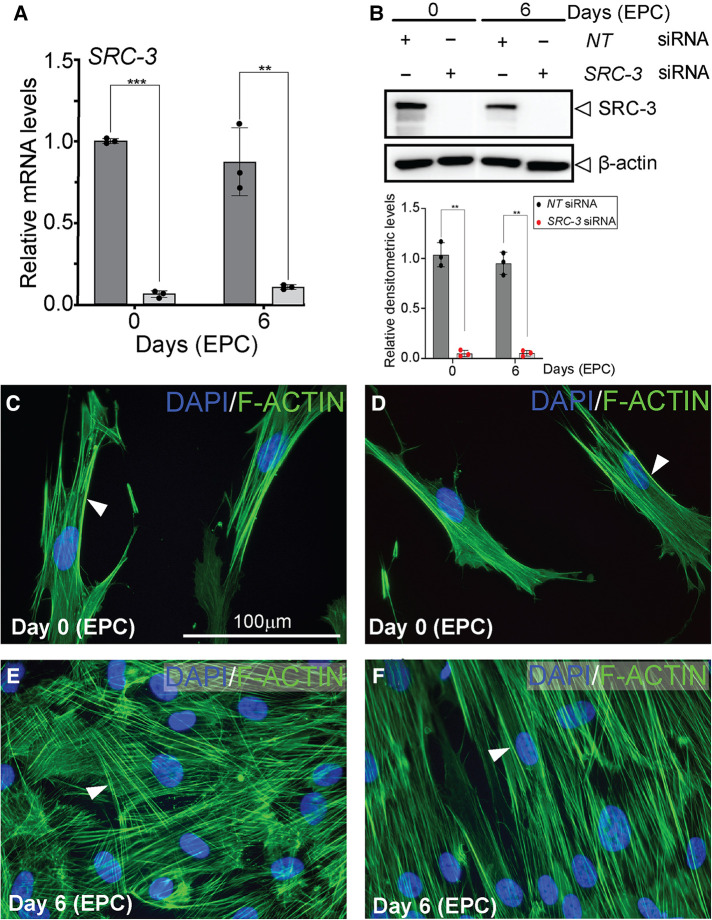
Decidualization of T-HESCs requires SRC-3 expression (**A**) siRNA mediated knockdown effectively reduces *SRC-3* levels in T-HESCs both at day 0 and day 6 of EPC culture. The histogram's dark grey bar on the left indicates T-HESCs transfected with *NT* siRNAs whereas the light grey bars on the right denote T-HESCs transfected with *SRC-3* siRNAs. Note the persistent knockdown of *SRC-3* from day 0 to day 6 of EPC culture. (**B**) Top panel shows that the western data confirm the results shown in (**A**). Note the significant knockdown of SRC-3 protein to undetectable levels following transfection with siRNAs targeted to *SRC-3* at day 0 and 6 of EPC treatment; β-actin serves as a loading control. Bottom panel shows that ImageJ analysis quantitatively confirms the western data shown in the top panel. The black and red dots for each bar in the histogram represent the number of technical replicates per time point/treatment group. Error bars denote a mean average of densitometric intensity per time point/treatment group ± standard deviation (SD); ** denote a *p*-value ≤ 0.01. (**C,D**) show T-HESCs at day 0 of EPC treatment that are transfected with siRNAs targeting *NT* or *SRC-3* respectively. Cells were immunofluorescently stained with DAPI (blue nuclei) and phalloidin [green fibers; binds F-actin (arrowhead)]. Note the typical parallel arrangement of F-actin filaments in the pre-decidual fibroblastic T-HESCs at this early stage of EPC culture. (**E**) and (**F**) display T-HESCs at day 6 EPC treatment, which were transfected with siRNAs targeting *NT* and *SRC-3* respectively. Note in (**E**) the expected spatial disorganized pattern of F-actin filaments in *NT* siRNA transfected T-HESCs as cells transform from a fibroblastoid to an epithelioid decidual cell morphology following six days of EPC culture. (**F**) Under similar culture conditions in (**E**), T-HESCs transfected with siRNAs targeting *SRC-3* fail to show this F-actin disorganized pattern and continue to display the ordered F-actin parallel pattern as observed in the pre-decidual stromal fibroblast shown in [**D** (white arrowhead)]. Scale bar in (**C**) applies to (**D–F**).

Although SRC-3 is expressed in HESCs, the observation that SRC-3 levels do not significantly change following decidualization raises the question: Is SRC-3 functionally required for HESC decidualization? To address this question, we used an established T-HESC line (48–50), which has been extensively used to study numerous factors implicated in HESC decidualization (39, 51–54). Analysis by qPCR revealed that siRNA mediated silencing significantly reduced *SRC-3* RNA levels in T-HESCs and resulted in near undetectable levels of SRC-3 protein, even after day 6 of EPC culture (Figures 3A,B). Importantly, *SRC-3* knockdown in T-HESCs did not significantly change SRC-1 or SRC-2 expression at the RNA or protein level (Supplementary Figure S3). This result is important as we have demonstrated that SRC-2 is essential for decidualization of cultured HESCs as well as murine endometrial decidualization (55, 56). Conversely, we found that *SRC-1* is not required for T-HESC decidualization (data not shown). At the cell morphology level (Figures 3C–F), *SRC-3* knockdown resulted in the inability of T-HESCs to transform from a fibroblastoid to an epithelioid morphology following administration of the deciduogenic hormone cocktail (EPC). For these cell-based studies, phalloidin fluorescent detection of F-actin was used (39). Isolated from the “death cap” mushroom (*Amanita phalloides*), phalloidin irreversibly binds juxtaposed actin molecules within the F-actin filament assembly. In the case of *NT* siRNA knockdown, T-HESCs change cellular morphology from a pre-decidual fibroblastic cellular shape with an ordered parallel organization of F-actin filaments (39) to a polygonal shape that is typical of the decidual cell, underscored by a striking disorganized arrangement of F-actin filaments (Figures 3C,E). With *SRC-3* siRNA knockdown, however, F-actin filaments remain parallel in T-HESCs following EPC treatment (Figures 3D,F), indicating that depletion of SRC-3 impairs the ability of T-HESCs to adopt the cellular morphology that characterizes a decidualized endometrial stromal cell (Figures 3D,F). Confirming the above qPCR and western results (Figures 3A,B), negligible levels of SRC-3 were immunofluorescently detected in T-HESCs following *SRC-3* siRNA transfection (Supplementary Figure S4).

**Figure 4:**
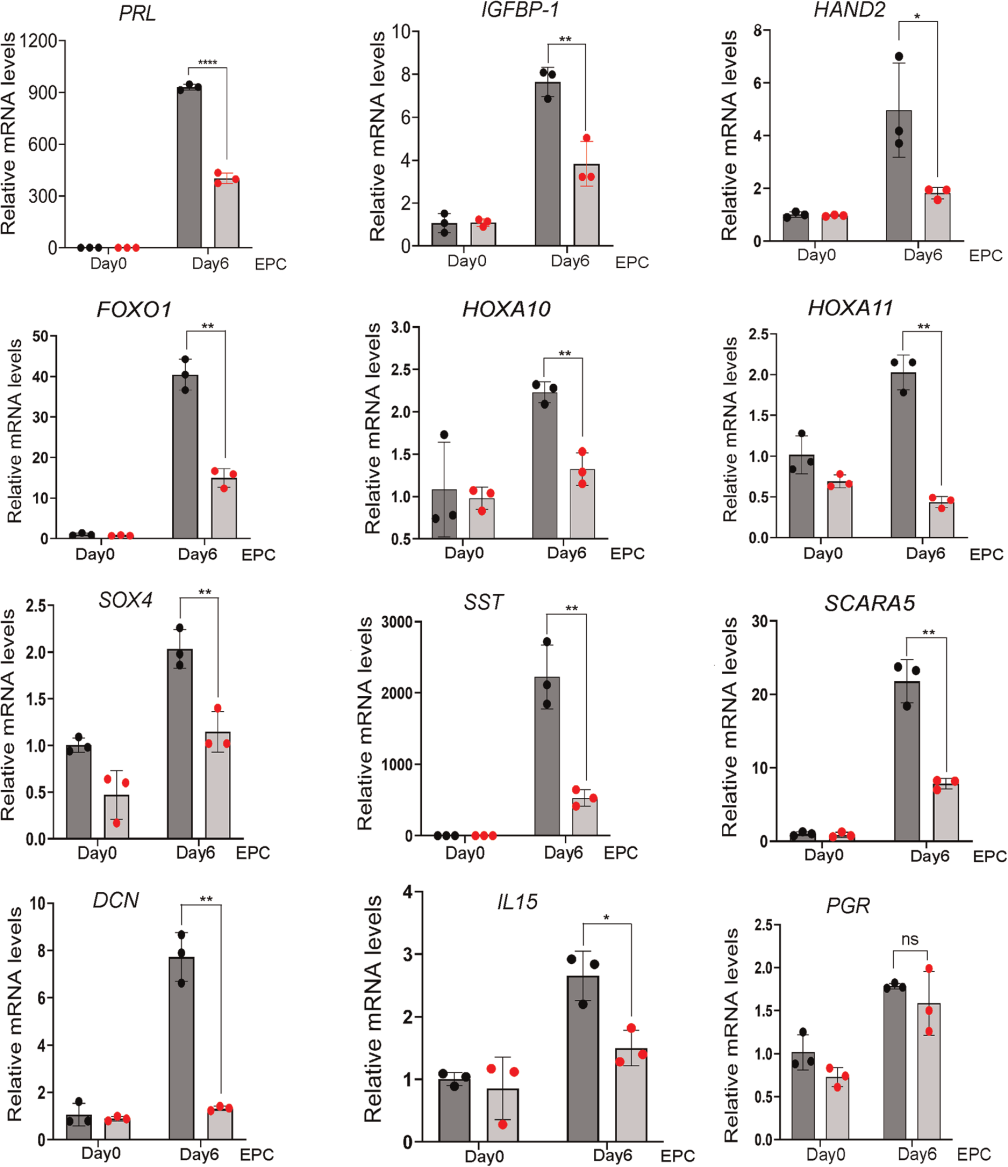
Depletion of *SRC-3* in T-HESCs results in a significant attenuation in the induction of established decidual cell markers following EPC culture. Quantitative real-time PCR reveals that siRNA mediated *SRC3* knockdown resulted in a marked reduction in the induction of the following established molecular targets: *PRL*, prolactin; *IGFBP-1*, insulin growth factor binding protein-1; *HAND2*, heart and neural crest derivative expressed 2; *FOXO1*, forkhead box 1; *HOXA10*, homeobox A10; *HOXA11*, homeobox A11; *SOX4*, SRY-Box transcription factor 4; *SST*, somatostatin; *SCARA5*, scavenger receptor class A member 5; *DCN*; decorin; *IL15*, interleukin 15; and *PGR*, progesterone receptor.

The above cell morphology results were supported at the molecular level by the significant reduction by *SRC-3* knockdown in T-HESCs in the induction of a gene subset that has been previously documented as important for decidualization or for cellular functions of the decidual cell (Figure 4). These genes include: prolactin [*PRL* (42)]; insulin like growth factor binding protein-1 [*IGFBP1* (57)]; heart and neural crest derivatives expressed 2 [*HAND2* (58, 59)]; forkhead box 1 [*FOXO1* (12, 60)]; homeobox A10 [*HOXA10* (61)]; homeobox A11 [*HOXA11* (62)]; SRY-Box transcription factor 4 [*SOX4* (63)],; somatostatin [*SST* (64)], and scavenger receptor class A member 5 [*SCARA5* (65)]; DECORIN [*DCN* (39)]; interleukin 15 *[IL15* (66)*]*; and *PGR*, progesterone receptor (67). Interestingly, *PGR* [and *WNT4* (68, 69) (data not shown)] levels did not change with *SRC-3* knockdown, indicating that a decrease in *PGR* levels is not the mechanism by which the decidual induction of the above genes is changed as a result of *SRC-3* depletion, and that not all genes associated with decidualization are affected by SRC-3 depletion.

### Transcriptomic analyses of T-HESCs depleted of SRC-3 prior to receiving the deciduogenic stimulus

**Figure 5:**
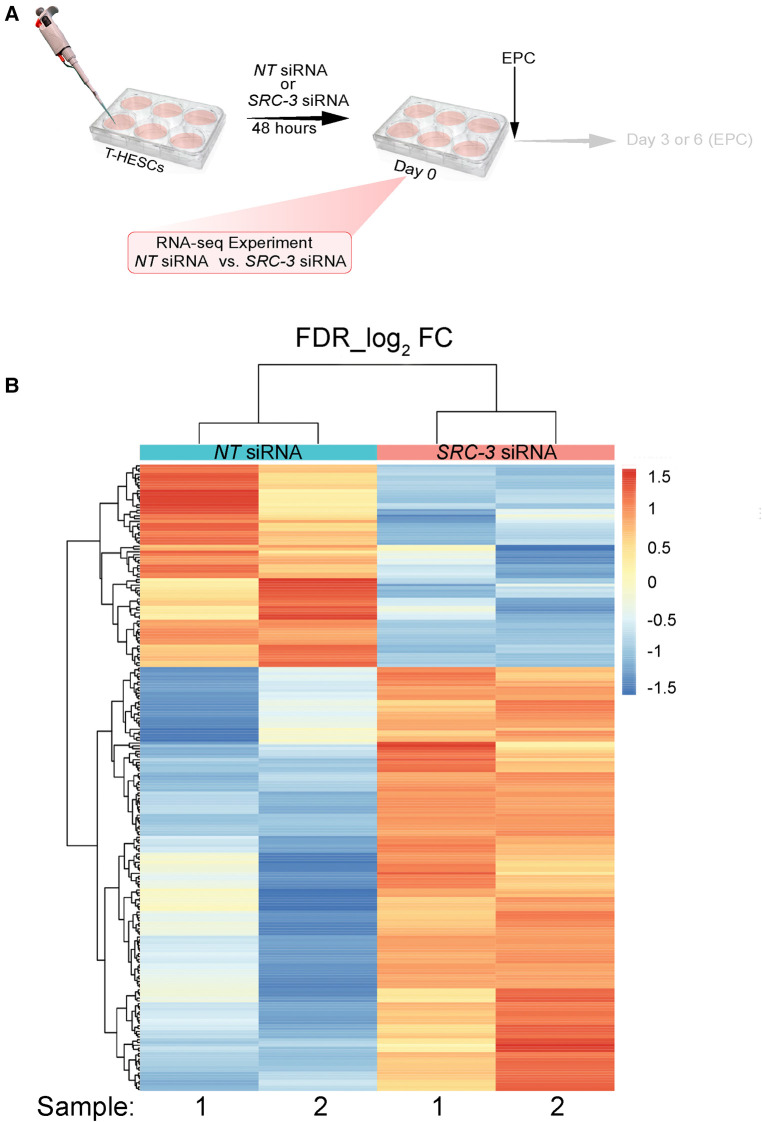
The SRC-3 dependent transcriptome in T-HESCs prior to receiving the deciduogenic hormone stimulus (**A**) the experimental design of the RNA-seq experiment showing that T-HESCs were transfected with *NT* or *SRC-3* siRNAs for forty-eight hours prior to being harvested for total RNA isolation; note: T-HESCs did not receive the EPC medium. Cells were harvested from two wells of a six well plate for each replicate; two replicates per *NT* and *SRC-3* siRNA treatment group were used for these RNA-seq experiments. (**B**) Heatmap of clustered genes with the same expression level differentially expressed (up or down) between the *NT* siRNA and *SRC-3* siRNA groups. Using a FDR <0.05 and an IFCI > 1.5 cutoff, 226 genes were differentially expressed (73 down and 153 up) between the *NT* and *SRC-3* siRNA groups. The 226 genes were clustered and displayed as a heat map, in which each horizontal row represents a single gene. Warmer and cooler colors (*i.e.* reds and blues respectively) represent higher and lower expression respectively: the vertical color key on the right indicates the color intensity with normalized expression values.

Given SRC-3 is required for T-HESC decidualization (Figures 3, 4), RNA-seq analysis was performed to determine whether the T-HESC transcriptome is significantly changed as a result of SRC-3 depletion. Such a transcriptome change is predicted to compromise the T-HESC's ability to correctly respond to the deciduogenic signal and execute normal functions of a decidual cell. The overall RNA-seq experimental design is shown (Figure 5A). Briefly, T-HESCs were transfected with *NT* siRNA (control) or *SRC-3* siRNAs for forty-eight hours. Instead of switching to the deciduogenic EPC medium, transfected cells (at day 0 EPC) were processed for RNA-seq analysis (Figure 5A).

Duplicate samples per treatment group were used for the RNA-seq experiment; cells were isolated from two wells of a six-well plate per sample. The complete list of genes differentially expressed between the two groups is reported in an Excel sheet in the supplementary section (Supplementary Folder S1). A total of 795 (499 upregulated and 296 downregulated) expressed genes were identified by RNA-seq were significantly changed (Supplementary Folder S1); 153 upregulated and 73 downregulated genes in this gene set reached the predetermined FDR (≤0.05) and FC (≥1.5) cutoffs. The FKPM values for all 795 genes were analyzed by PCA [Supplementary Folder S1 (PCA tab)]. The PCA showed that the *NT* siRNA- and *SRC-3* siRNA-treatment groups were significantly separated in terms of their respective duplicates. Tables 2, 3 list the top 50 genes down and up regulated respectively that met the FDR (≤0.05) and FC (≥1.5) cutoffs whereas the expression heatmap (Figure 5B) shows the top 226 genes (153 upregulated and 73 downregulated) between the *NT* siRNA and *SR-3* siRNA treatment groups. The above RNA-seq datasets were deposited in the NCBI GEO repository (GEO accession number: GSE210936).

**Table 2:** Top 50 downregulated genes with > 2 log2FC and ≤ 0.05 FDR in *SRC-2* knockdown THESCs line.

GENE SYMBOL	GENE ID	GENE NAME	log2FC
CCN2	1,490	cellular communication network factor 2	−9.65 × 10^102^
H4C3	8,364	H4 clustered histone 3	−2.56 × 10^24^
INHBA	3,624	inhibin subunit beta A	−7.00 × 10^6^
AMIGO2	347,902	adhesion molecule with Ig like domain 2	−264,526
H2BC11	8,970	H2B clustered histone 11	−3112.61
PEA15	8,682	proliferation and apoptosis adaptor protein 15	−2324.81
H2AC21	317,772	H2A clustered histone 21	−2322.8
VAMP2	6,844	vesicle associated membrane protein 2	−1162.73
H4C4	8,360	H4 clustered histone 4	−840.643
RFLNB	359,845	refilin B	−558.069
RGS4	5,999	regulator of G protein signaling 4	−452.524
PSME3	10,197	proteasome activator subunit 3	−243.068
ZFP36L2	678	ZFP36 ring finger protein like 2	−226.152
SMIM10	644,538	small integral membrane protein 10	−166.513
PURA	5,813	purine rich element binding protein A	−132.028
H3C7	8,968	H3 clustered histone 7	−49.0812
C11orf68	83,638	chromosome 11 open reading frame 68	−37.1825
UACA	55,075	uveal autoantigen with coiled-coil domains and ankyrin repeats	−34.6828
RPN2	6,185	ribophorin II	−17.2915
IGIP	492,311	IgA inducing protein	−13.4947
H3C1	8,350	H3 clustered histone 1	−11.9711
H2BC6	8,344	H2B clustered histone 6	−11.8612
TMED10	10,972	transmembrane p24 trafficking protein 10	−11.8282
FOXC2	2,303	forkhead box C2	−9.9483
TMEM200A	114,801	transmembrane protein 200A	−8.96914
CAMK2N1	55,450	calcium/calmodulin dependent protein kinase II inhibitor 1	−8.49212
EIF2S3	1,968	eukaryotic translation initiation factor 2 subunit gamma	−8.42607
MAPK1IP1l	93,487	mitogen-activated protein kinase 1 interacting protein 1 like	−7.82586
PORCN	64,840	porcupine O-acyltransferase	−6.91333
CCN3	4,856	cellular communication network factor 3	−6.27663
SCARNA9l	100,158,262	small Cajal body-specific RNA 9 like	−6.16203
FERMT2	10,979	FERM domain containing kindlin 2	−5.33035
SF3B4	10,262	splicing factor 3b subunit 4	−4.9419
H3C10	8,357	H3 clustered histone 10	−4.92587
RNF11	26,994	ring finger protein 11	−4.71412
TCEAL7	56,849	transcription elongation factor A like 7	−4.35703
FOXL1	2,300	forkhead box L1	−4.04063
DIMT1	27,292	DIM1 rRNA methyltransferase and ribosome maturation factor	−3.36136
TCAF1	9,747	TRPM8 channel associated factor 1	−3.19254
DCAKD	79,877	dephospho-CoA kinase domain containing	−3.17144
ARRDC3	57,561	arrestin domain containing 3	−3.15592
ASNSD1	54,529	asparagine synthetase domain containing 1	−3.11208
FYTTD1	84,248	forty-two-three domain containing 1	−3.09781
GTF3C6	112,495	general transcription factor IIIC subunit 6	−3.00018
PNPO	55,163	pyridoxamine 5′-phosphate oxidase	−2.63178
DTX3l	151,636	deltex E3 ubiquitin ligase 3l	−2.62797
ATMIN	23,300	ATM interactor	−2.59801
USP53	54,532	ubiquitin specific peptidase 53	−2.49268
AP2B1	163	adaptor related protein complex 2 subunit beta 1	−2.38829
REEP3	221,035	receptor accessory protein 3	−2.37707

**Table 3:** Top 50 upregulated genes with > 5 log2FC and ≤ 0.05 FDR in *SRC-2* knockdown THESCs line.

GENE SYMBOL	GENE ID	GENE NAME	log2FC
TUBA1B	10,376	tubulin alpha 1b	2.94 × 10^137^
GAPDH	2,597	glyceraldehyde-3-phosphate dehydrogenase	8.54 × 10^45^
TMSB10	9,168	thymosin beta 10	1.28 × 10^38^
CCN1	3,491	cellular communication network factor 1	9.64 × 10^37^
TUBB	203,068	tubulin beta class I	1.55 × 10^32^
MRFAP1	93,621	Morf4 family associated protein 1	6.04 × 10^29^
TIMP1	7,076	TIMP metallopeptidase inhibitor 1	1.43 × 10^12^
PTMA	5,757	prothymosin alpha	2.80 × 10^7^
RPS5	6,193	ribosomal protein S5	1.90 × 10^7^
HSPD1	3,329	heat shock protein family D (Hsp60) member 1	2.46 × 10^6^
SRM	6,723	spermidine synthase	363,539
EIF4A1	1,973	eukaryotic translation initiation factor 4A1	294,764
FLNC	2,318	filamin C	30,075.5
PPP1R14B	26,472	protein phosphatase 1 regulatory inhibitor subunit 14B	1757.37
CCT5	22,948	chaperonin containing TCP1 subunit 5	615.43
KIF20A	10,112	kinesin family member 20A	590.523
KLF6	1,316	Kruppel like factor 6	540.256
UCP2	7,351	uncoupling protein 2	275.479
PSMC3	5,702	proteasome 26S subunit, ATPase 3	223.144
VCP	7,415	valosin containing protein	221.506
H2AX	3,014	H2A.X variant histone	146.018
ID3	3,399	inhibitor of DNA binding 3, HLH protein	123.636
TOMM22	56,993	translocase of outer mitochondrial membrane 22	103.895
CALM1	801	calmodulin 1	44.3066
NGRN	51,335	neugrin, neurite outgrowth associated	41.6473
ARL6IP4	51,329	ADP ribosylation factor like GTPase 6 interacting protein 4	37.8062
BIRC5	332	baculoviral IAP repeat containing 5	28.7831
SOD1	6,647	superoxide dismutase 1	27.475
TPM3	7,170	tropomyosin 3	23.4603
HRAS	3,265	HRas proto-oncogene, GTPase	21.8272
TYMS	7,298	thymidylate synthetase	20.5197
PDZD11	51,248	PDZ domain containing 11	19.2009
PSMC1	PSMC1	proteasome 26S subunit, ATPase 1	18.7238
HDGF	3,068	heparin binding growth factor	17.647
FLOT1	10,211	flotillin 1	17.0194
CENPF	1,063	centromere protein F	12.8586
TUBA1C	84,790	tubulin alpha 1c	12.6358
FLII	2,314	FLII actin remodeling protein	12.3552
TRIP6	7,205	thyroid hormone receptor interactor 6	11.4362
CALM3	808	calmodulin 3	10.8634
RAD23A	5,886	RAD23 homolog A, nucleotide excision repair protein	9.7879
NES	10,763	nestin	9.33474
TACC3	10,460	transforming acidic coiled-coil containing protein 3	9.18109
ADRM1	11,047	ADRM1 26S proteasome ubiquitin receptor	8.78648
TSR3	115,939	TSR3 ribosome maturation factor	8.31174
PSMA4	5,685	proteasome 20S subunit alpha 4	8.22639
ENSA	2,029	endosulfine alpha	7.76648
SMC1A	8,243	structural maintenance of chromosomes 1A	7.67597
IK	3,550	IK cytokine	6.87798
JPT1	51,155	Jupiter microtubule associated homolog 1	6.62842

**Figure 6:**
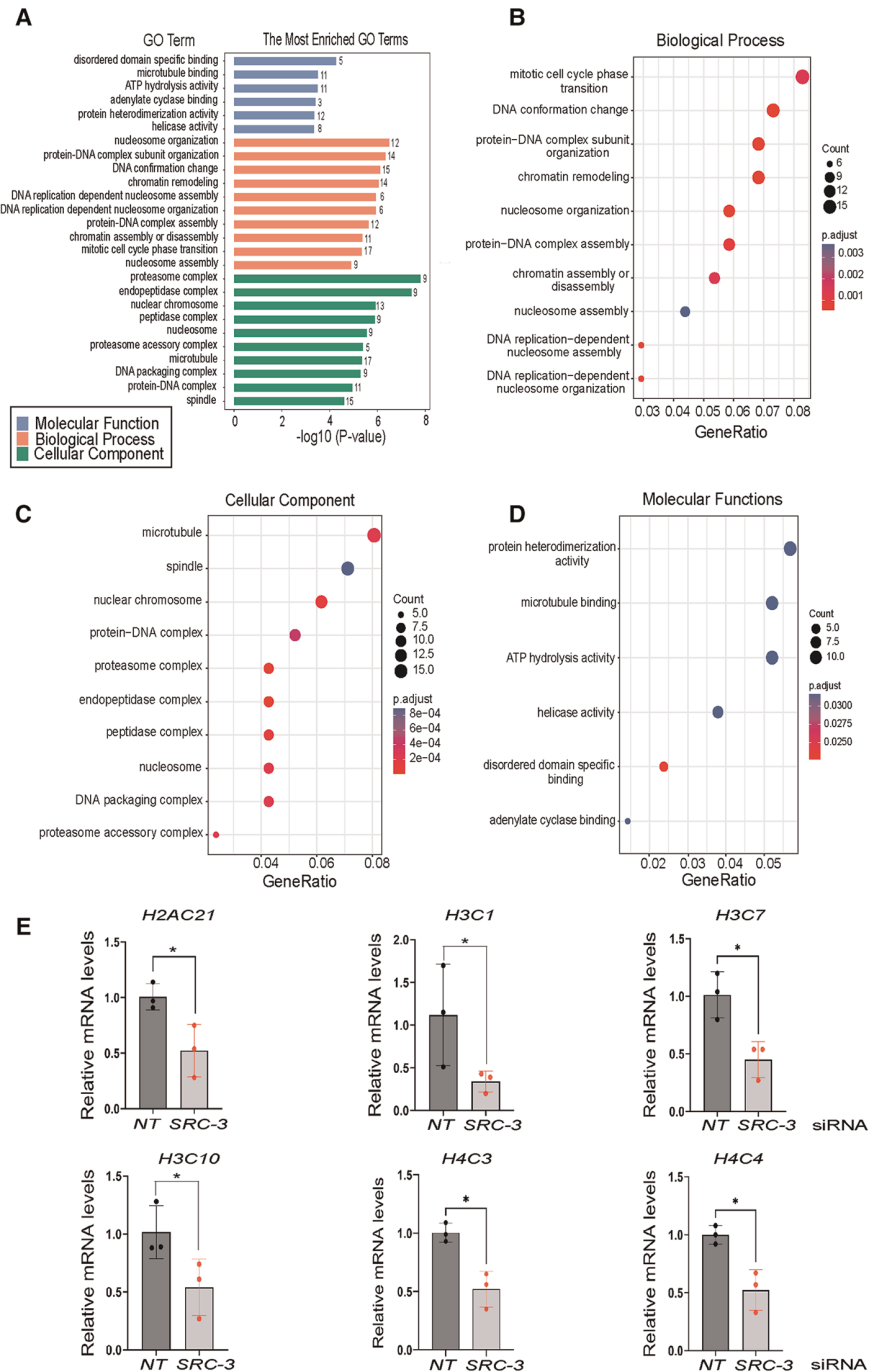
Gene ontology functional analyses of differentially expressed genes in T-HESCs following *SRC-3* knockdown (**A**) gene ontology (GO) enrichment analysis of differentially expressed genes was achieved using the DAVID. The six significantly enriched GO terms (*p* < 0.05) in molecular function along with the ten top significantly enriched GO terms in biological processes and cellular component branches are presented. The adjusted *p*-values of the terms were –log10 transformed. (**B–D**) Dot plots of enriched genes within the differentially expressed gene set are stratified according to biological processes, cellular components, and molecular functions respectively. (**E**) Quantitative real time PCR analysis shows significant reduction in the induction of the following histone family members: *H2AC21*, *H3C1*, *H3C7*, *H3C10*, *H4C3*, and *H4C4* in T-HESCs following *SRC-3* knockdown.

Using the agglomeration and gene ontology (GO) tools in DAVID, genes in the differentially expressed gene set were grouped according to GO terms, which were further stratified into the following biological modules: biological processes, cellular components, and molecular functions (Figures 6A–D). Our analyses revealed a significant enrichment in the differentially expressed gene dataset for genes involved in mitotic phase cycle transition, chromatin remodeling, nucleosome organization, and DNA replication dependent nucleosome assembly (Figures 6A–D). Related to the above, the use of KEGG (Kyoto Encyclopedia of Genes and Genomes) pathway annotation software showed that protein families involved in genetic information processing scored the highest in terms of number of genes assigned to a given biological process (Supplementary Figure S5). Interestingly, a review of the differential gene expression table (Supplementary Folder S1) uncovered a significant overrepresentation of genes encoding members of the core histone class of proteins, a subset of which was validated by qPCR (Figure 6E).

**Figure 7:**
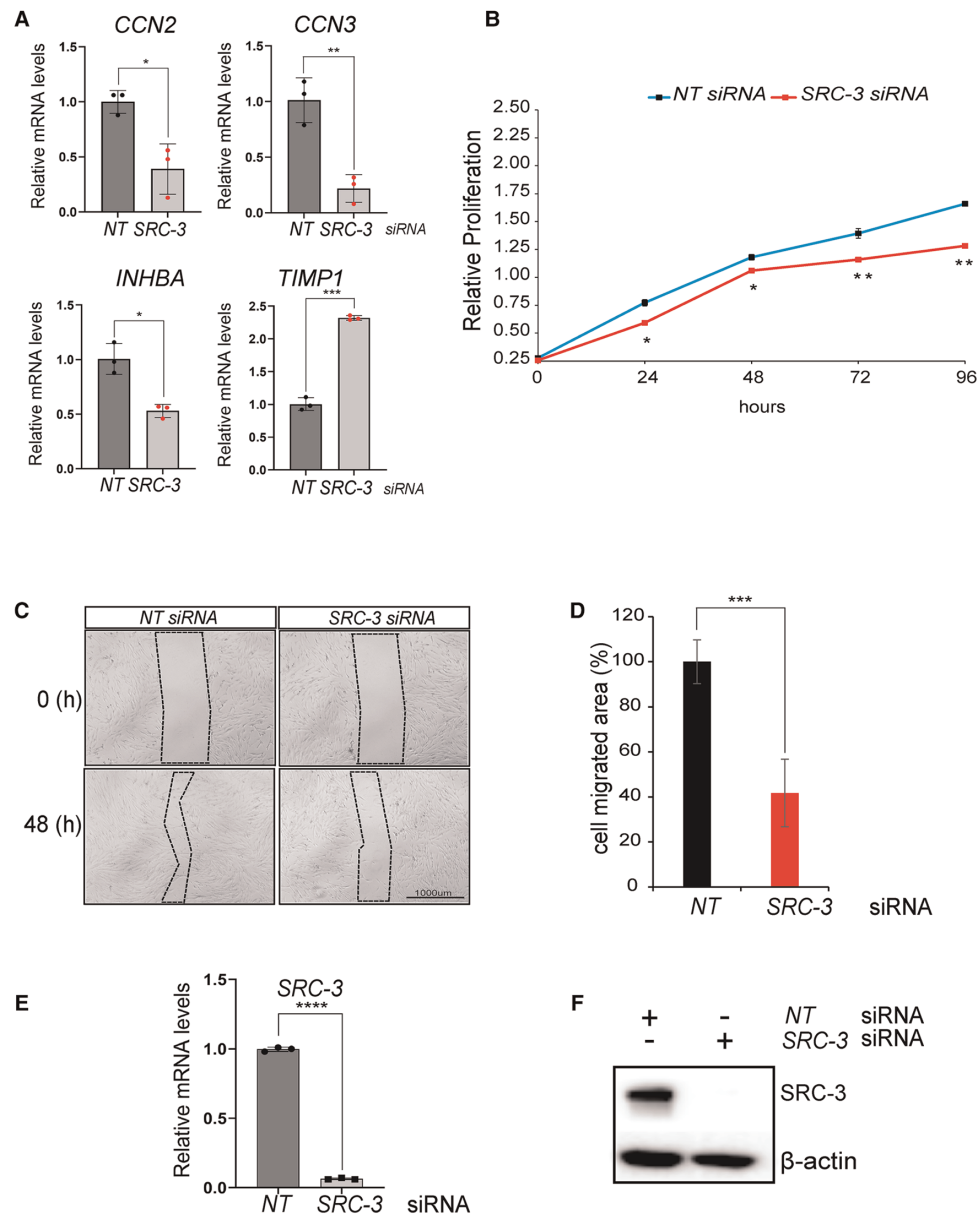
Proliferative and migratory properties of T-HESCs are significantly reduced following *SRC-3* knockdown. (**A**) Quantitative real-time PCR analyses shows a significant reduction in the induction of *CCN2*, *CCN3*, *INHBA*, and *TIMP1* following *SRC-3* knockdown. (**B**) The MTT assay demonstrates that depletion of *SRC-3* levels in T-HESCs markedly reduces this cell type's ability to maintain optimum cell viability/ proliferative capacity over time. (**C**) The wound healing assay demonstrated that *SRC-3* knockdown resulted in a significant reduction in the migratory abilities of T-HESCs. Shown is a representative bright-field image of the migrated area (demarcated by dotted line) forty eight hours following the application of the scratch to the cell monolayer, previously transfected with either *NT* or *SRC-3* siRNAs; scale bar applies to all images. (**D**) The histogram quantitatively displays the reduced migratory ability of T-HESCs following *SRC-3* knockdown. Migratory ability of T-HESCs is reduced by at least 50% following *SRC-3* knockdown (compare black bar (*NT* siRNA) with red bar (*SRC-3* siRNA)). (**E,F**) Quantitative real time PCR and western immunoblot analyses confirm efficient *SRC-3* knockdown in T-HESCs at the RNA and protein level respectively in these experiments. Results in (**E**) are displayed as ± SE and are representative of three independent experiments; *p-value <0.05; **p-value <0.01; ***p-value <0.001; and ****p-value < 0.0001. Western in (**F**) is representative of three separate experiments; β-actin was used as a loading control.

Genes that are involved in cell proliferation, migration, and invasion were also enriched in the differentially expressed gene dataset (Figure 7A). For example, the Cell Communication Network (CCN) family of cysteine-rich matricellular proteins are extracellular matrix (ECM)-associated proteins that are active in a wide spectrum of biologies and pathobiologies (70). In particular, the CCN2 and CCN3 matricellar proteins underpin numerous cellular activities that range from mitogenesis, differentiation, survival, adhesion, migration, chemotaxis, angiogenesis, chondrogenesis, and wound healing (71, 72). Accordingly, CCN2 and CCN3 dysregulation is causal for a multitude of human pathobiologies, including tumorigenesis and metastasis (73, 74). Interestingly, these proteins have been implicated in female reproductive disorders, such as preeclampsia (75–77); however, their role in the normal endometrium is unclear. Also linked to cellular proliferation, migration and invasion in other physiologies, inhibin beta A (also known as INHBA) was originally recognized as a subunit for the closely related activin and inhibin glycoproteins, which exert opposing functional effects. However, INHBA perturbation alone has been associated with aggressive tumor behavior, including acceleration of cell proliferation, epithelial mesenchymal transition (EMT), migration and invasion (78–80). Noteworthy, INHBA has been detected in the human endometrium and endometrial pathologies (81, 82); however, the role of INHBA in the endometrial stromal cell remains an open question. In addition to inhibiting matrix metalloproteinases, tissue inhibitor of metalloproteinases 1 (TIMP1) can signal in a cytokine-like manner to influence numerous biological processes, which includes cellular proliferation, differentiation, apoptosis, angiogenesis, and oncogenesis (83–85). Although detected in murine and human endometrial tissue, TIMP1's role in endometrial function is currently unknown.

Although functionally validating these new genes in HESC decidualization is beyond the scope of the current study, the various cellular properties attributed to these genes provided the impetus to test whether SRC-3 controls these cell activities, specifically cell viability, migration, and invasive properties of HESCs, especially as SRC-3 controls these cellular functions in other physiologies and pathologies (86–88). To address the aforementioned, the MTT assay demonstrated that SRC-3 is necessary for full proliferative capacity of the T-HESC, and that the necessity for SRC-3 increases as the duration of culture lengthens (Figure 7B). In addition, SRC-3 is also essential for the known intrinsic migratory (Figures 7C–F) and invasive properties of endometrial cells (Supplementary Figure S6), cellular properties that are essential for a fully functional decidua *in vivo* (89–92).

## Discussion

Originally discovered as an oncogenic coactivator (93), SRC-3 [also known as amplified in breast cancer 1 (AIB1)] is now recognized as a pivotal coactivator for a broad spectrum of physiological processes, ranging from metabolism, circadian rhythms to T cell biology (18, 94–96). Importantly, SRC-3 is implicated in female reproductive physiology and pathophysiology (24).

In the case of endometrial pathology, Lessey's group reported that SRC-3 levels (along with SRC-2 [also known as transcriptional intermediary factor 2 (TIF2)] are markedly elevated in epithelial and stromal cells of secretory-phase endometrial tissue biopsied from patients diagnosed with polycystic ovary syndrome (PCOS) (40). Apart from an increased susceptibility for endometrial cancer (97), PCOS patients are predisposed to additional reproductive sequelae, which include low cycle fecundity and a high miscarriage rate that can reach 60%–70% (98, 99). Independent studies have also shown that SRC-3 levels are strikingly elevated in hyperplastic and/or malignant human endometrial tissue (100–104).

In agreement with previous studies (40), we show here that SRC-3 is expressed in the glandular epithelial and stromal compartments of a healthy endometrium, and that endometrial SRC-3 expression does not significantly alter with cycle stage or with changes in hormone exposure (i.e., from an E2 to an E2 plus P4 environment). In the case of cultured T-HESCs, we found that neither SRC-3's spatial distribution nor expression level markedly changed during decidualization. Notable, however, endometrial SRC-3 levels are aberrantly elevated in a number of gynecological morbidities, which include endometrial hyperplasia and cancer (100, 101), PCOS (40), and endometriosis (105). Here, our bioinformatic analysis also showed that abnormally elevated levels of SRC-3 occur in endometrial tissue biopsied from patients diagnosed with RPL (Figure 2F). Therefore, these findings furnish tantalizing support for the proposal that unscheduled elevation of endometrial SRC-3 levels may serve as a biomarker for the emergence of these uterine pathobiologies. Interestingly, SRC-3's phosphorylation status in T-HESCs is altered between the pre-decidual and decidualized state. Specifically, the levels of SRC-3, which harbored phosphorylated serine residue 857 (S857), increased in decidual cells compared with pre-decidual cells. As phosphorylation of SRC-3 at residue S857 is known to modulate and extend coactivator potency in other physiological systems (22, 106, 107), this PTM event may signal a change in SRC-3 activation status between the pre-decidual and decidual cell. Future investigations will test whether this PTM (as well as other PTMs singly or in combination) is functionally important for SRC-3's role in decidualization and/or in the functional properties of the decidual cell following its development from a HESC progenitor.

By depleting pre-decidual HESCs of total SRC-3 protein, we demonstrated that SRC-3 is essential for these cells to transform into specialized decidual cells; the decidual defect could not be compensated by other SRC members. Importantly, the normal induction of the majority molecular signals tested—hormones, growth factors, transcription factors, and cytokines, which have been shown to be functionally important for this cellular transformation process (or for correct functioning of the decidual cell once formed), was significantly attenuated in response to diminished SRC-3 levels. Collectively, these results underscore the importance of SRC-3 in sustaining the pivotal transcriptional responses that manifest in HESCs, which ensure HESC development to a normally functioning decidual cell. Because the EPC-induction of transcription factors [i.e., *HAND2* (58, 66)] and paracrine signals [i.e., *IL15* (12, 66, 108–111)] is significantly attenuated in SRC-3 depleted HESCs, SRC-3 is required for optimum HESC intra- and extracellular signaling.

The derailment in the induction of these and other known transcriptional programming events in HESCs with a SRC-3 deficit provided the pretext to identify the early HESC molecular signals and associated biological processes that are compromised following reduction of SRC-3 levels. Specifically, the focus of the transcriptomic analysis was to determine the extent to which SRC-3 depletion would alter the transcriptome of the pre-decidual cell prior to receipt of the deciduogenic stimulus.

Significant reduction of SRC-3 levels in pre-decidual HESCs over a forty eight hour period resulted in a marked change in the transcriptome that normally exists at a time when these cells receive the deciduogenic hormone stimulus. Gene enrichment analysis revealed that the expression of genes involved in chromatin remodeling, organization and assembly of the nucleosome and control of DNA replication was significantly reduced in HESCs with reduced SRC-3 levels. Noteworthy was the significant reduction in the expression levels of core histone variants (i.e., *H3AC21*; *H3C1*; *H3C7*; *H3C10*; *H4C3*; and *H4C4*). From yeast to humans, histone homeostasis is essential for avoiding genomic stress, changes in global transcriptional output, premature replicative senescence and ageing (112–123). Normally, strict controls on histone gene expression levels are in place during the cell cycle to ensure direct coupling of DNA replication with canonical histone deposition (124–126). Interestingly, however, scheduled increases in histone content are associated with cellular differentiation and ploidy, both cellular processes that underpin HESC decidualization (127). Following SRC-3 knockdown, the reduction in the expression levels of core histone variants, along with other factors implicated in nucleosome organization and assembly, may in part contribute to the observed decrease in HESC proliferative capacity as cellular proliferation followed by differentiation is a requirement for completion of the decidualization process (7).

It should be noted that the above findings from our cell-based investigations have yet be confirmed by *in vivo* model systems. Female mice, which are deficient in SRC-3, exhibit dwarfism, delayed pubertal onset, attenuated mammary gland morphogenesis, striking metabolic impairments, and a severe subfertility defect (128). Because of the phenotypic complexity displayed by the whole body *SRC-3* knockout mouse, we recently generated a conditional *SRC-3* knockout mouse by crossing our *Pgr-cre* driver mouse with a mouse carrying the floxed SRC-3 allele (129, 130). Interestingly, the conditional *SRC-3* knockout female mouse is infertile, and its endometrium fails to decidualize (unpublished data), supporting the *in vitro* studies described here.

In conclusion, our studies offer compelling support for an important role for SRC-3, which is independent of the other SRC family members, in HESC decidualization. Devoid of SRC-3, HESCs are less viable and lose significant proliferative, differentiative, motile, and invasive capabilities, cellular attributes that are necessary for either the development or function of the decidual cell. Given decidualization is critical for advancement of the implantation process toward placentation, further investigation of endometrial SRC-3 in periimplantation biology is predicted to furnish new molecular insights not only into normal endometrial function but also endometrial dysfunction that leads to early pregnancy loss.

## Data Availability

The datasets presented in this study can be found in online repositories. The names of the repository/repositories and accession number(s) can be found in the article/**Supplementary Material**.

## Glossary

AAALAC, association for the assessment and accreditation of laboratory animal care; AD, activation domain; AIB1, amplified in breast cancer 1; ANOVA, analysis of variance; ATCC, american type culture collection; BHLH, basic helix loop helix; CCN2, cellular communication network factor 2; CCN3, cellular communication network factor 3; DAPI, 4′,6′-diamidino-2-phenylindole; DAVID, database for annotation, visualization, and integrated discovery; DCN, decorin; DMEM, Dulbecco's modified eagle's medium; DMSO, dimethyl sulfoxide; E2, estradiol; ECM, extracellular matrix; EMT, epithelial mesenchymal transition; EPC, 17β-estradiol, medroxyprogesterone acetate, 2′-O-dibutyryladenosine-3′, 5′ cAMP; EPL, early pregnancy loss; FC, Fold change; FDR, false discovery rate; FOXO1, forkhead box O1A; FPKM, fragments per kilobase of exon per million mapped fragments; GE, glandular epithelium; GEO, gene expression omnibus; GSEA, gene set enrichment analysis; GO, gene ontology; HAT, histone acetyl transferase; H3AC21, H3A clustered histone 21; H3C1, H3 clustered histone 1; H3C7, H3 clustered histone 7; H3C10, H3 clustered histone 10; H4C3, H4 clustered histone 3; H4C4, H4 clustered histone 4; HAND2, heart- and neural crest derivatives-expressed protein 2; HESC, human endometrial stromal cell; HOXA, Homeobox A; HRP, horse radish peroxidase; IACUC, institutional animal care and use committee; IL15, Interleukin 15; IGFBP-1, insulin like growth factor binding protein-1; INHBA, inhibin beta A; IRB, Institutional review board; ITS-A, insulin, transferrin, selenite and sodium pyruvate; LXXLL, Leucine-X-X-leucine-leucine; KEGG, kyoto encyclopedia of genes and genomes; MSIGDB, molecular signatures database; MTT, 3-(4, 5-dimethylthiazol-2-yl)-2, 5-diphenyltetrazolium bromide; NCBI, national center for biotechnology information; NCOA, Nuclear coactivator; NT, Non targeting; P4, Progesterone; P300/CBP, EP300/CREB-binding protein; PAS, Per-Arnt-Sim; PBS, phosphate buffered saline; PCA, principal component analysis; PCOS, polycystic ovary syndrome; PGR, progesterone receptor; PGR-CRE, progesterone receptor cre; PRL, prolactin; PTMs, posttranslational modifications; PVDF, polyvinylidene difluoride; RID, receptor interaction domain; RIN, RNA integrity number; RNA-seq, RNA sequencing; RPL, recurrent pregnancy loss; Q, polyglutamine; QPCR, quantitative polymerase chain reaction; SCARA5, Scavenger receptor class A member 5; Scid, Severe combined immunodeficiency; SD, Standard deviation; SDECs, singly dispersed endometrial cells; SDS-PAGE, sodium dodecyl sulfate polyacrylamide; SE, standard error; SFBS, stripped fetal bovine serum; siRNA, small interfering RNA; SOX4, SRY-box transcription factor 4; SST, somatostatin; SRC-1, steroid receptor coactivator-1; SRC-2, steroid receptor coactivator-2; SRC-3, Steroid receptor coactivator-3; STR, short tandem repeat; TBS-T, tris-buffered saline with Tween 20; T-HESCs, Telomerase-transformed human endometrial stromal cells; TIF-2, Transcription intermediary factor; TIMP1, Tissue inhibitor of metalloproteinases 1; uNK, Uterine natural killer cells; WNT4, Wingless/integrated 4.
