# Supplementary figures and images for: Decidualization of human endometrial stromal cells requires steroid receptor coactivator-3
Source: Front Reprod Health. 2022 Nov 24;4:1033581. doi: 10.3389/frph.2022.1033581 (PMC9730893; doi:10.3389/frph.2022.1033581)

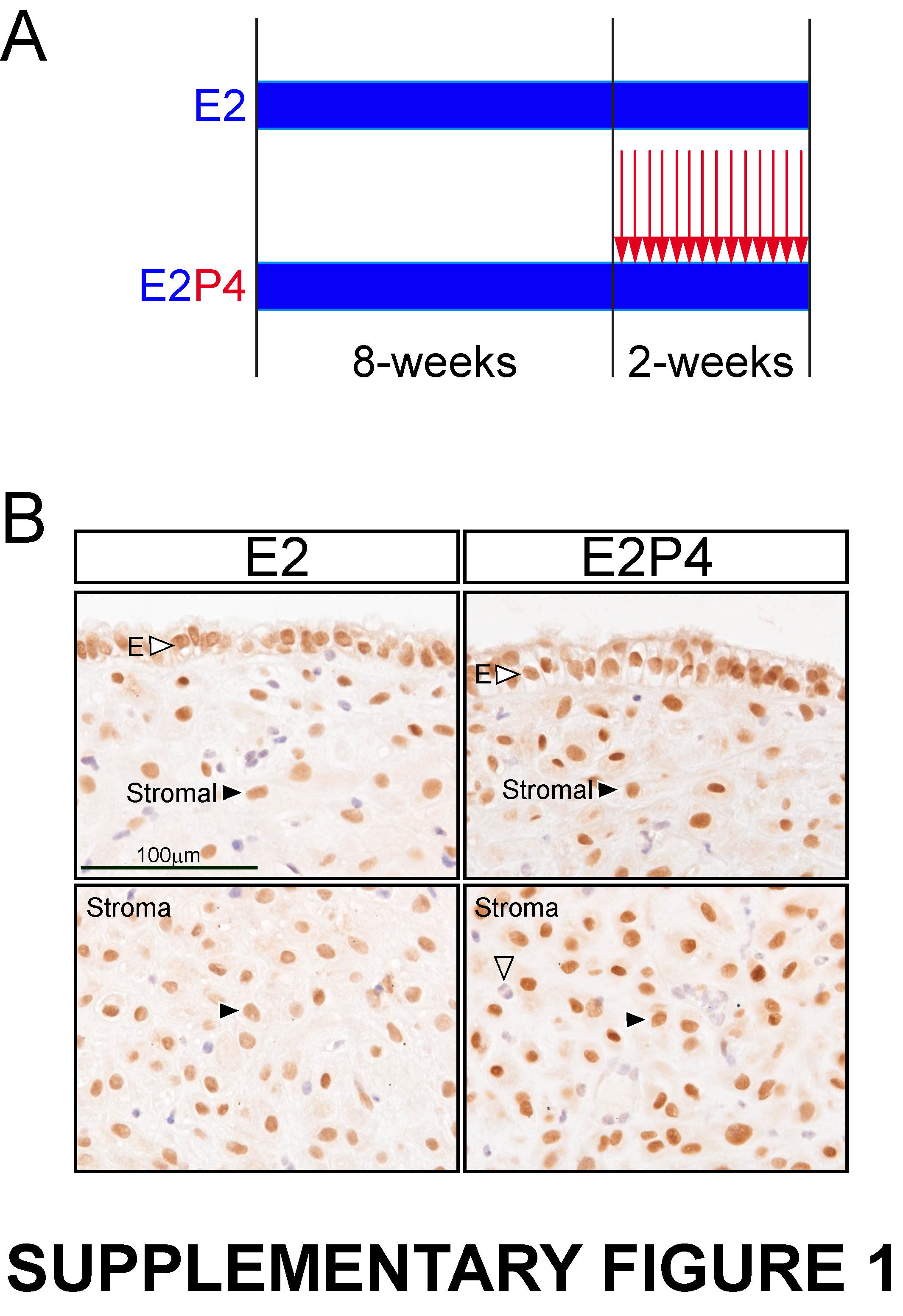

Supplement: Supplementary file 1 [file Image1.tif]

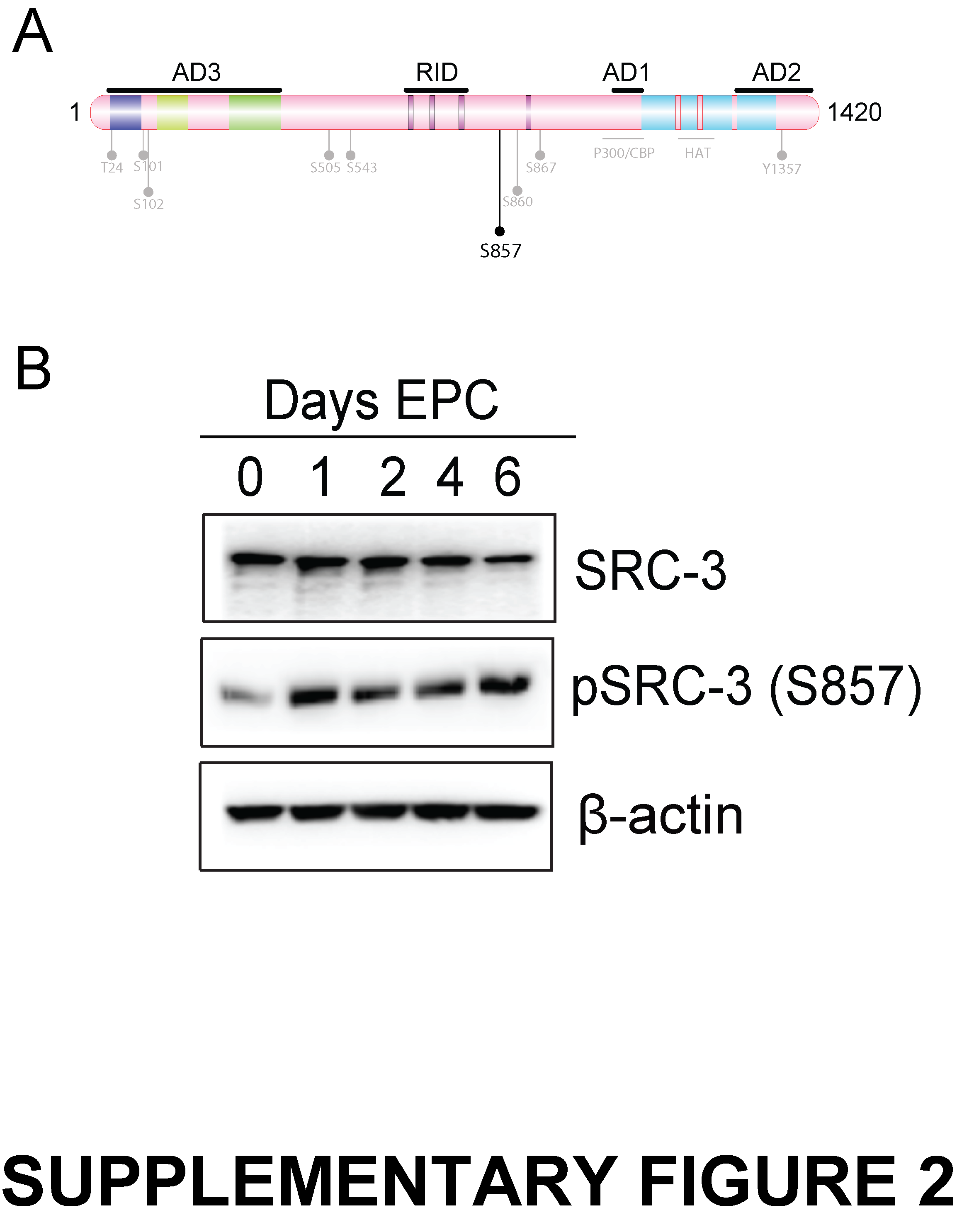

Supplement: Supplementary file 2 [file Image2.tif]

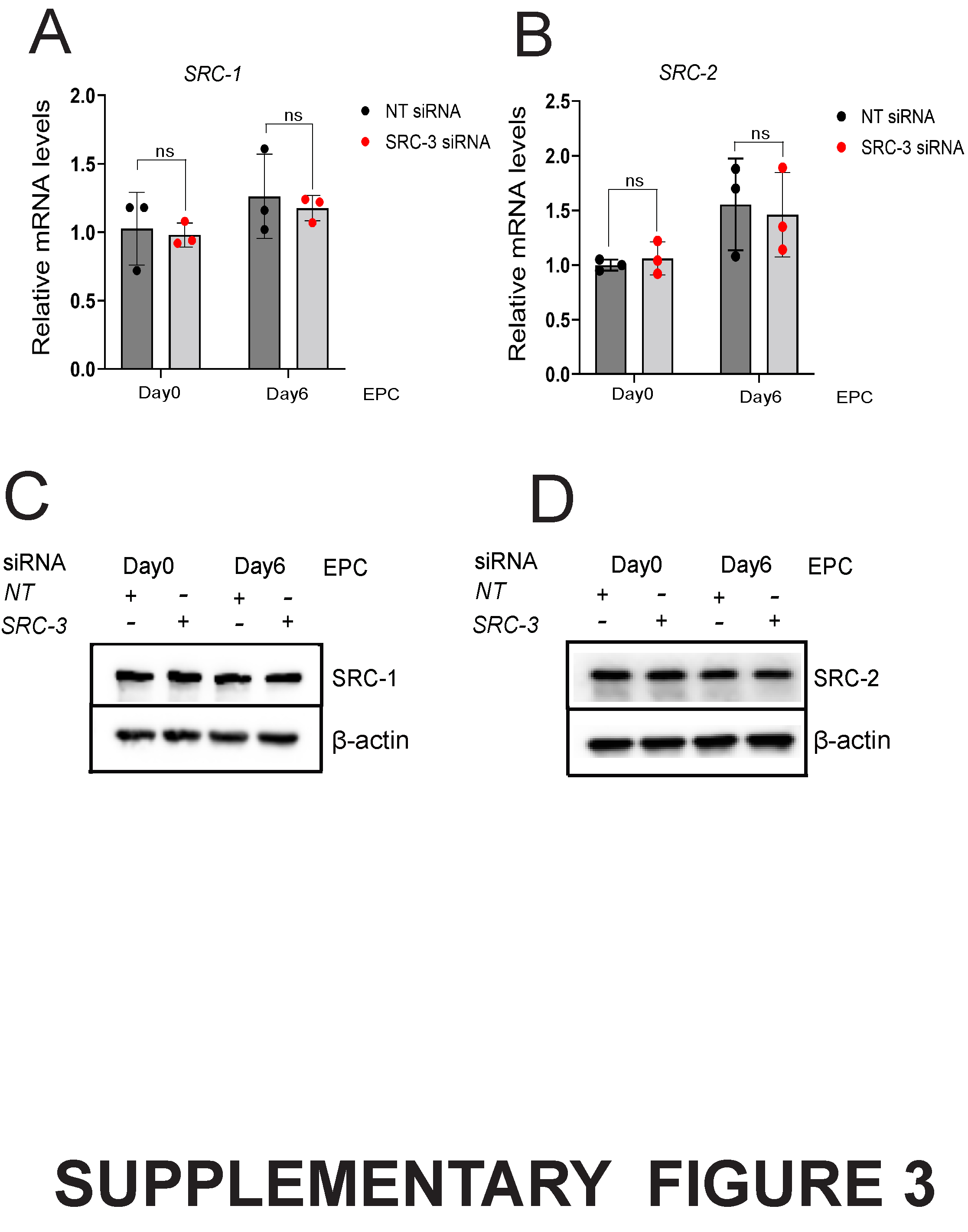

Supplement: Supplementary file 3 [file Image3.tif]

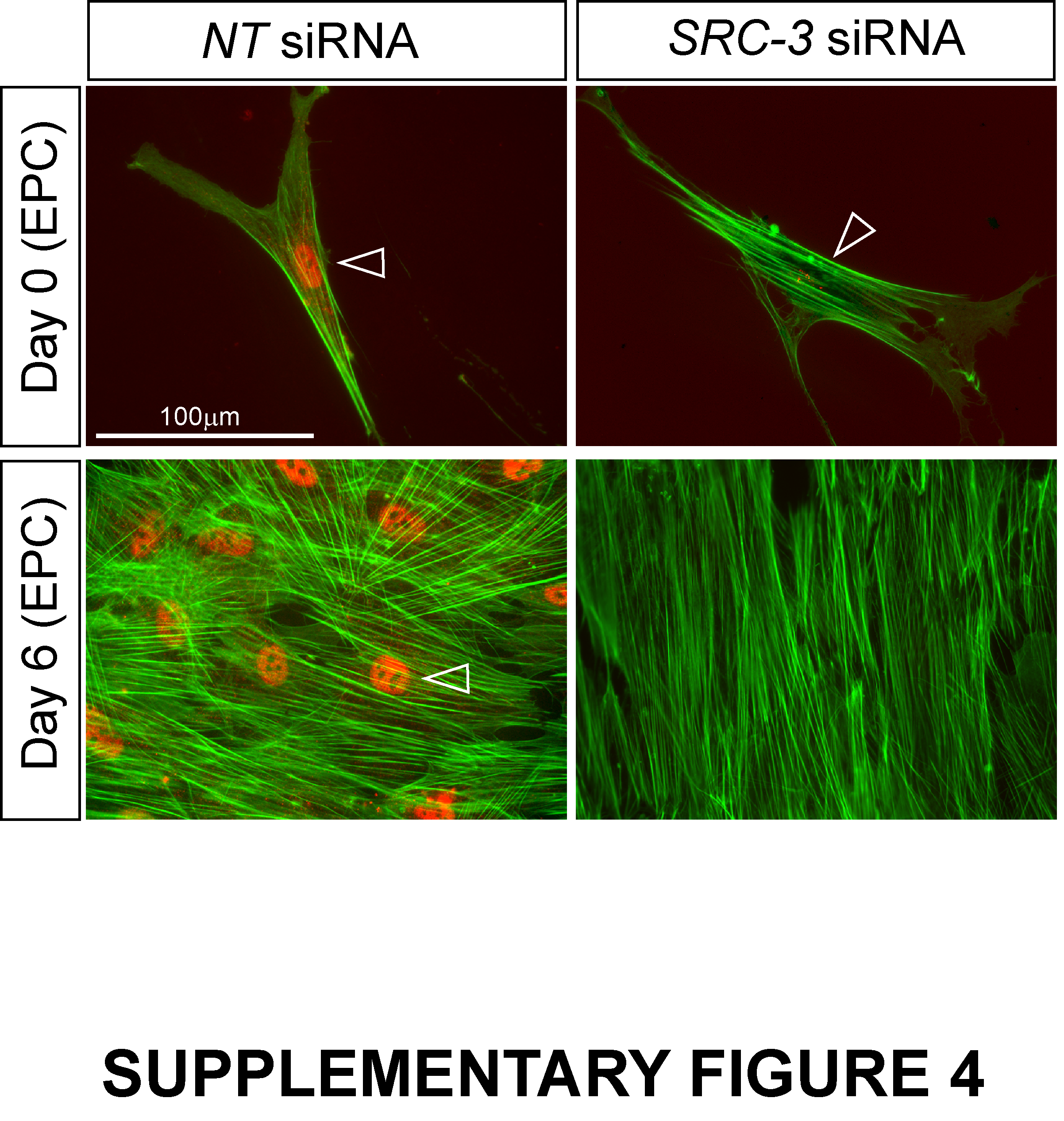

Supplement: Supplementary file 4 [file Image4.tif]

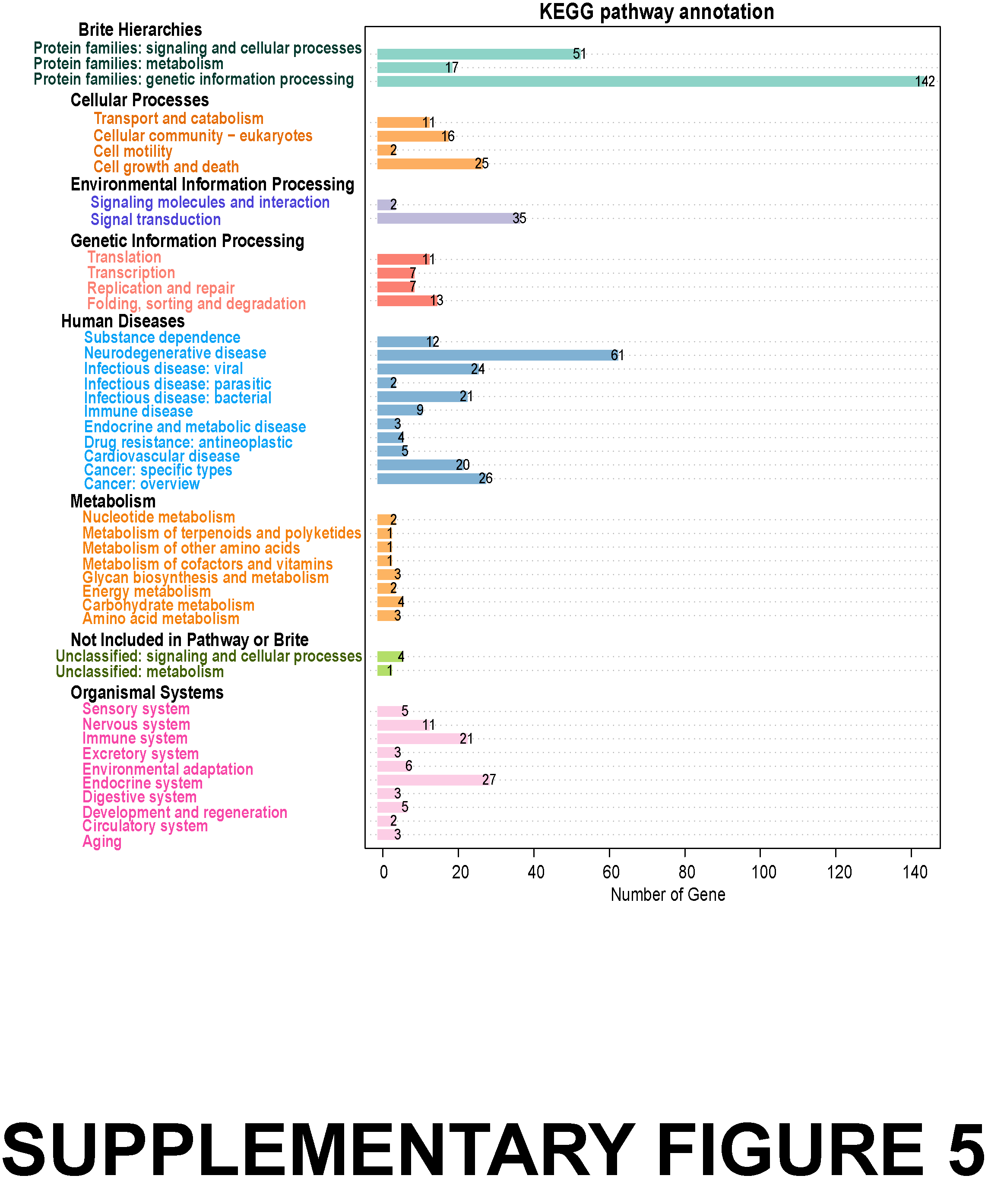

Supplement: Supplementary file 5 [file Image5.tif]

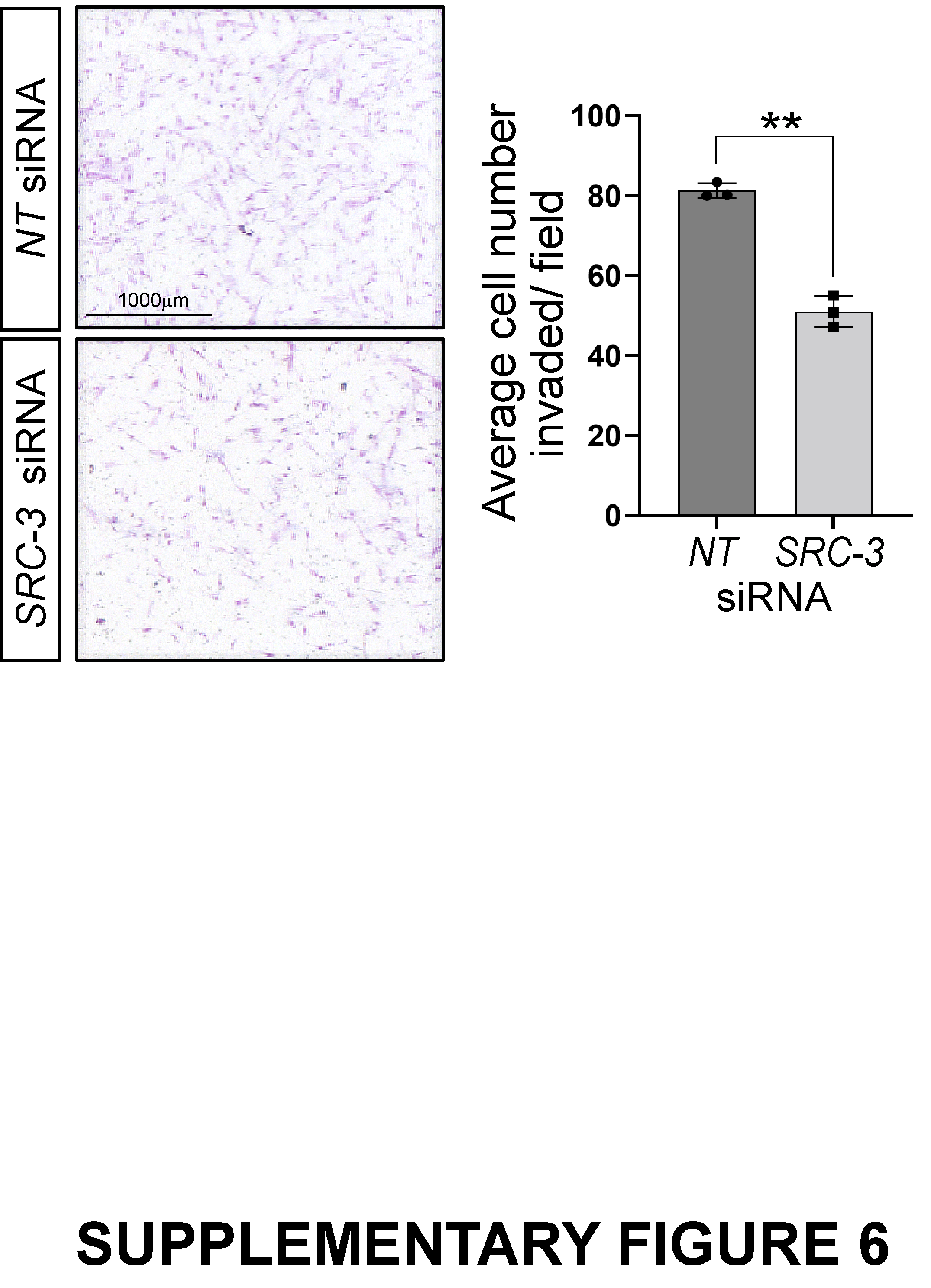

Supplement: Supplementary file 6 [file Image6.tif]
